# Catalogue of type specimens of *Sarcophaga* Meigen deposited in Shanghai Entomological Museum (Diptera, Sarcophagidae)

**DOI:** 10.3897/zookeys.1281.182881

**Published:** 2026-06-09

**Authors:** Chao Wang, Thomas Pape, Weibing Zhu, Li Dai, Qiyong Liu, Dong Zhang

**Affiliations:** 1 National Key Laboratory of Intelligent Tracking and Forecasting for Infectious Diseases, National Institute for Communicable Disease Control and Prevention, Chinese Center for Disease Control and Prevention, Beijing 102206, China Natural History Museum of Denmark, University of Copenhagen Copenhagen Denmark; 2 School of Ecology and Nature Conservation, Beijing Forestry University, Beijing 100083, China National Key Laboratory of Intelligent Tracking and Forecasting for Infectious Diseases, National Institute for Communicable Disease Control and Prevention, Chinese Center for Disease Control and Prevention Beijing China; 3 Natural History Museum of Denmark, University of Copenhagen, Universitetsparken 15, DK-2100, Copenhagen, Denmark School of Ecology and Nature Conservation, Beijing Forestry University Beijing China; 4 Center for Excellence in Molecular Plant Science, Chinese Academy of Sciences, Shanghai 200032, China Center for Excellence in Molecular Plant Science, Chinese Academy of Sciences Shanghai China

**Keywords:** Flesh flies, holotype, nomenclature, paratype, *
Sarcophaga
*, synonyms, syntype

## Abstract

A catalogue of the type specimens for nominal species-group taxa currently assigned to *Sarcophaga* deposited in the Shanghai Entomological Museum is presented. Type material of 22 of the 24 nominal species of *Sarcophaga* stated in the original paper to have type specimens deposited in the Shanghai Entomological Museum was recovered and examined, and these are documented with photographs for the first time. Two non-type specimens of *S. tsushimae* Senior-White, 1924 are shown to have been incorrectly labelled as paratypes but are included for completeness. No type material was recovered for the species Sarcophaga (Beziella) shenzhenensis (Fan, 2002) and Sarcophaga (Beziella) pudongensis (Fan, Chen & Lu, 2003). A syntype of *Sarcophaga parva* Quo, 1952 is shown to have been incorrectly labelled as holotype.

## Introduction

Shanghai Entomological Museum is a subunit of Shanghai Institute for Biological Sciences under the Chinese Academy of Sciences and is one of the major entomological research centers in China. It has valuable historical collections dating back to the Entomology Department of the Aurora Museum, which was founded by the French priest Pierre Marie Heude (Chinese name Shizen Han Bohu) in 1868 (Tai 2012). The Shanghai Entomological Museum is one of the major Chinese depositories for flesh fly type specimens, with the Zoological Museum, Institute of Zoology, Chinese Academy of Sciences, and the Academy of Military Medical Sciences, both located in Beijing, and it houses type specimens for almost 33% of the nominal species currently classified in *Sarcophaga* Meigen, 1826 and deposited in a Chinese institution (Wang 2020). Fan (1964, 1965, 1992) and Xue and Chao (1996) consulted the collections of the Shanghai Entomological Museum extensively for their work on Chinese flesh flies, but the flesh fly types of this museum have never been presented with thorough documentation. For this paper we examined all type specimens of nominal species currently classified in *Sarcophaga* and deposited in the Shanghai Entomological Museum, with photographs of habitus, terminalia, and labels for the first time.

## Materials and methods

Photographs were taken with a Canon 600D camera mounted on an Olympus SZX16 stereomicroscope. The stacked images were created in Helicon Focus 3.2 (Helicon Soft Ltd, Kharkov, Ukraine) and processed in Adobe Photoshop CS6 (Adobe Systems, Inc., San Jose, CA, USA). Terminalia were photographed in situ, and no dissections were made for the present study. Lectotypes were not designated from those syntypic series where no holotype had been designated, as none were found to be comprised of more than one species and therefore do not pose a threat to nomenclatural stability.

### Format and arrangement of the catalogue

Nominal species are listed alphabetically according to species epithet and numbered consecutively. Each nominal species is listed with a set of subheadings: “Name” giving the full name as given in the original work with author(s), year: page. “Type locality” is given in its English spelling and as detailed as possible based on the information in the original publication and the original label data (with Chinese words given in our translation). “Material examined” enumerates the type specimens, giving their type status, sex, and label data in standard notation. “Identity” gives the valid name of the taxonomic species as assessed in this work and following the generic and subgeneric classification of Pape (1996), Wang et al. (2020) and Pape and Whitmore (2022). “References” provides citations to the sources here considered to be the most important for the respective nominal species, arranged by the name used in the respective paper; it is not intended to be an exhaustive bibliography. “Remarks” lists the number and kind of types given in the original publication and provides other information considered important for historical, taxonomic, or nomenclatural reasons.

## Catalogue

**1. *abaensis***

Fig. 1

**Figure 1.**
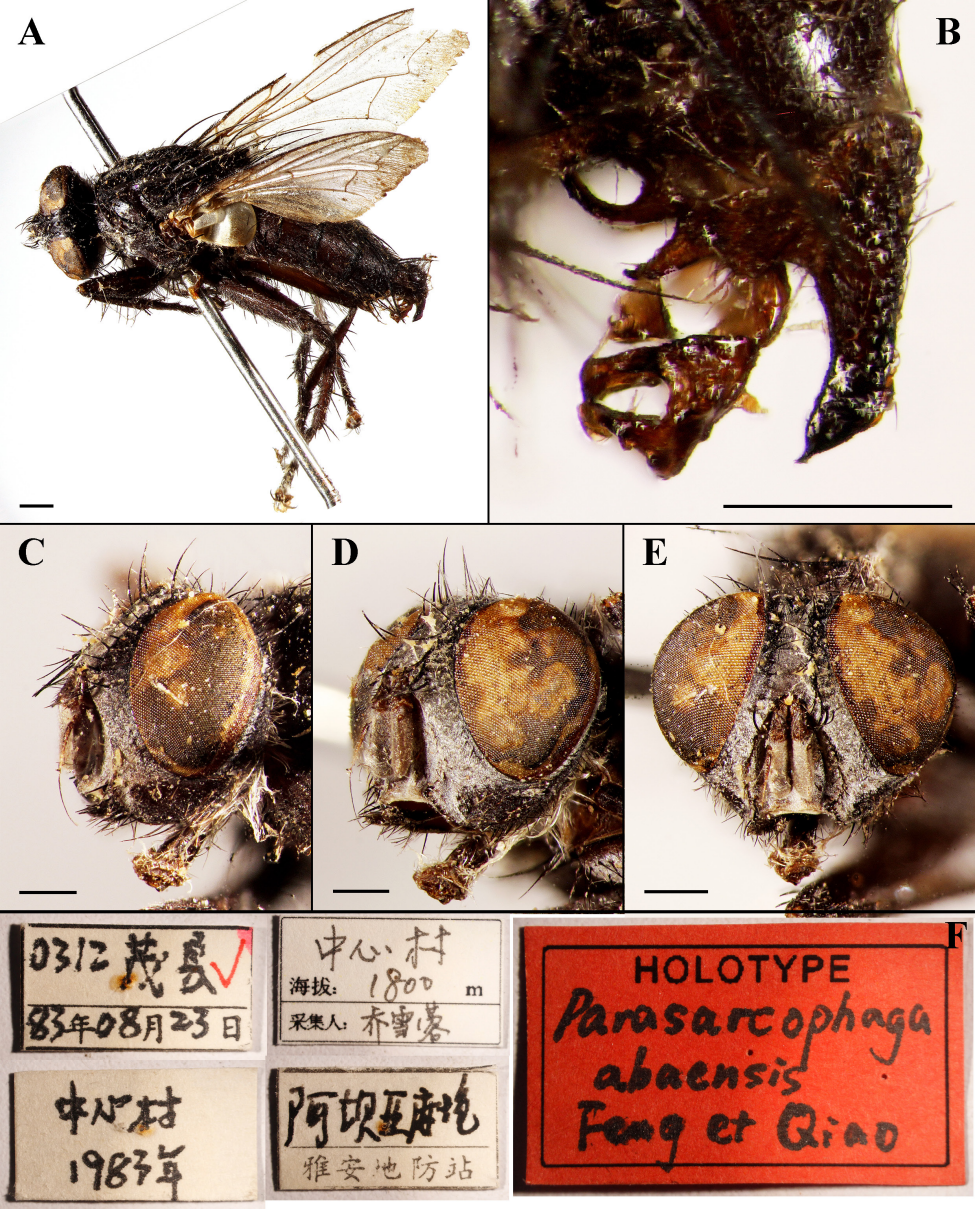
Holotype of *Parasarcophaga abaensis* Feng & Qiao, 2003, male. **A**. Body, lateral view; **B**. Terminalia, lateral view; **C**. Head, lateral view; **D**. Head, anterolateral view; **E**. Head, anterior view; **F**. Labels. Scale bars: 1 mm.

**Name**. *Parasarcophaga abaensis* Feng & Qiao, 2003: 267.

**Type locality**. China: Sichuan, Maoxian.

**Material examined**. Holotype (♂): Sichuan, Maoxian, 1800 m, 23.viii.1983, Xuerong Qiao leg. Paratypes: 2 males, same label data as holotype.

**Identity**. Sarcophaga (Parasarcophaga) abaensis (Feng & Qiao, 2003).

**References**. *Parasarcophaga abaensis*: Zhang (2014: 10), Verves (2020: 48).

Sarcophaga (Parasarcophaga) abaensis: Wang (2013: 34), Zhang (2014: 19, 50).

**Remarks**. The type series is composed of a male holotype, two male paratypes with same label data as holotype, and a male paratype from Luding, Luqiao. The latter was not recovered in the present study.

**2. affinis**

Fig. 2

**Figure 2.**
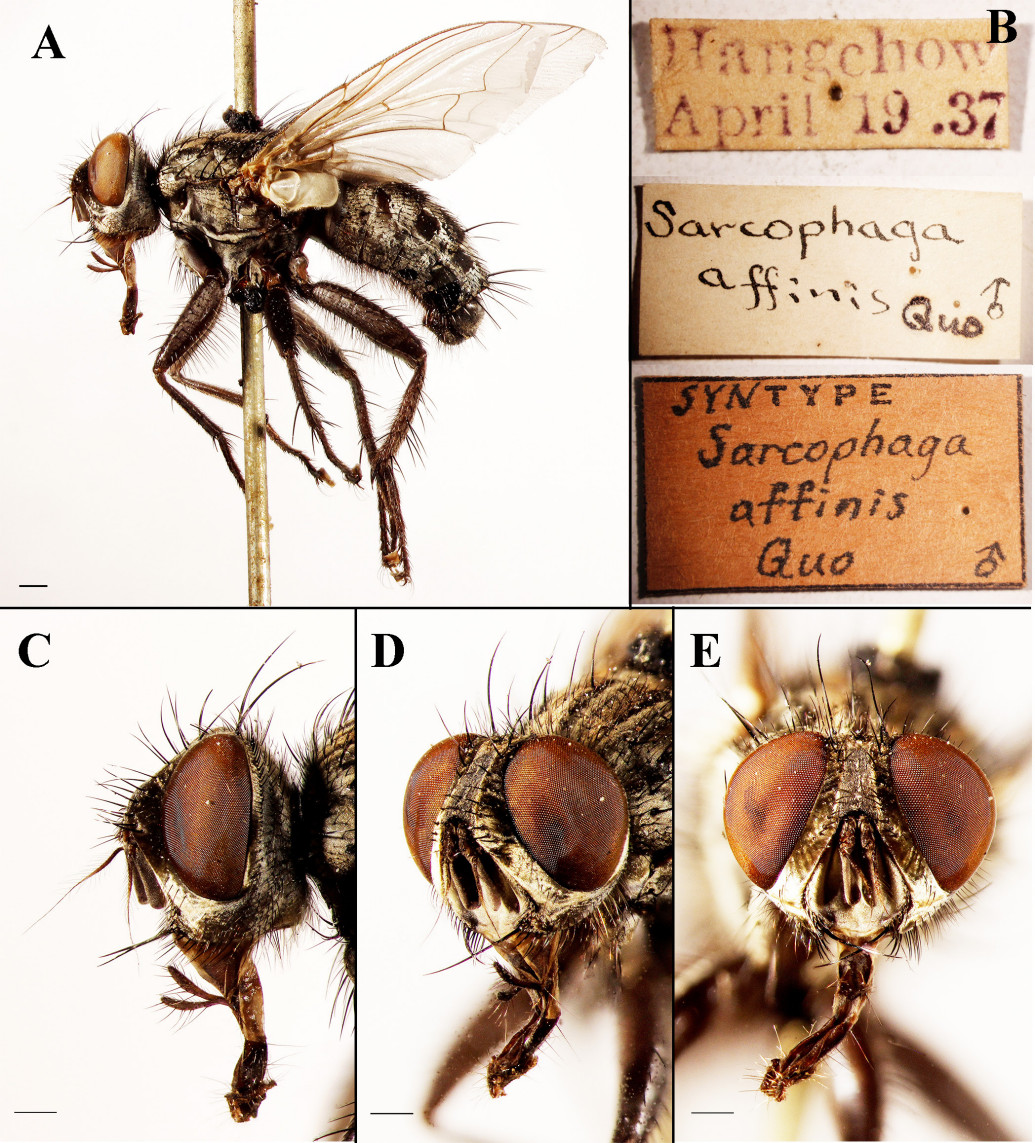
Syntype of *Sarcophaga affinis* Quo, 1952, male. **A**. Body, lateral view; **B**. Labels; **C**. Head, lateral view; **D**. Head, anterolateral view; **E**. Head, anterior view. Scale bars: 1 mm.

**Name**. *Sarcophaga affinis* Quo, 1952: 68.

**Type locality**. China: environs of Shanghai.

**Material examined**. Syntype (♂): Zhejiang, Hangzhou (Hangchow), 19.iv.1937, no further data.

**Identity**. Sarcophaga (Bellieriomima) pterygota Thomas, 1949.

**References**. *Bellieriomima pterygota*: Fan and Pape (1996: 248), Verves (2020: 33).

Pierretia (Bellieriomima) pterygota: Fan (1992: 676, 681), Xue and Chao (1996: 1629, 1634), Lei and Zhou (1998: 255), Fang and Wu (2001: 144), Xue and Tong (2001: 475), Yue (2016: 42, 102).

Pierreta (Bellieriomima) subulata pterygota: Fan (1965: 232, 236), Chao and Fan (1993: 610), Zhang et al. (1994: 282), Wei and Yang (2005: 424).

Sarcophaga (Bellieriomima) pterygota: Pape (1996: 301), Wang (2013: 28), Zhang (2014: 11, 26).

**Remarks**. The type series is composed of seven males, of which only one was recovered in the present study. Quo (1952) did not designate a holotype, which means that the other specimens are syntypes.

**3. *anchoriformis***

Figs 3, 4

**Figure 3.**
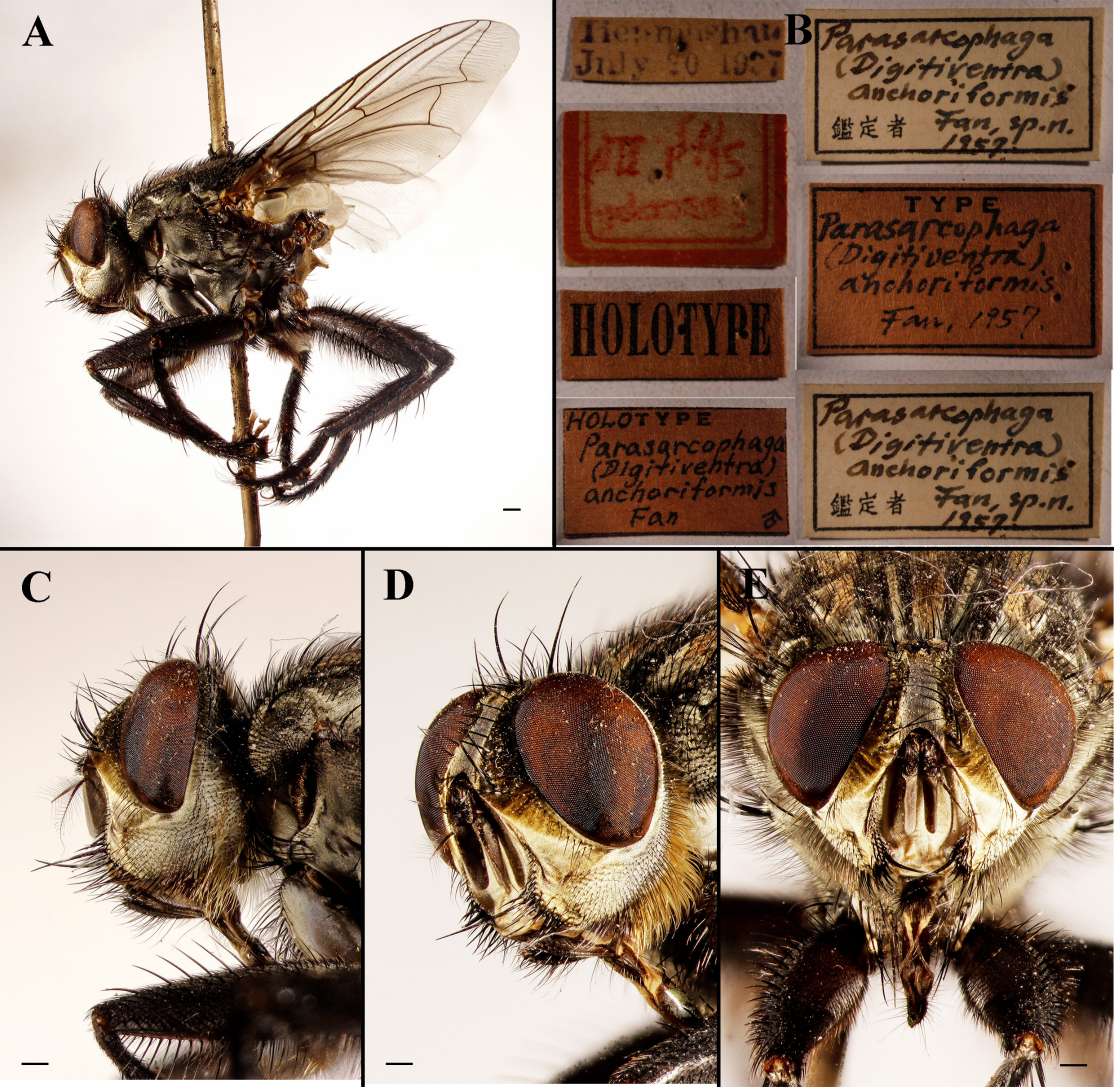
Holotype of Parasarcophaga (Digitiventra) anchoriformis Fan, 1964, male. **A**. Body, lateral view; **B**. Labels; **C**. Head, lateral view; **D**. Head, anterolateral view; **E**. Head, anterior view. Scale bars: 1 mm.

**Figure 4.**
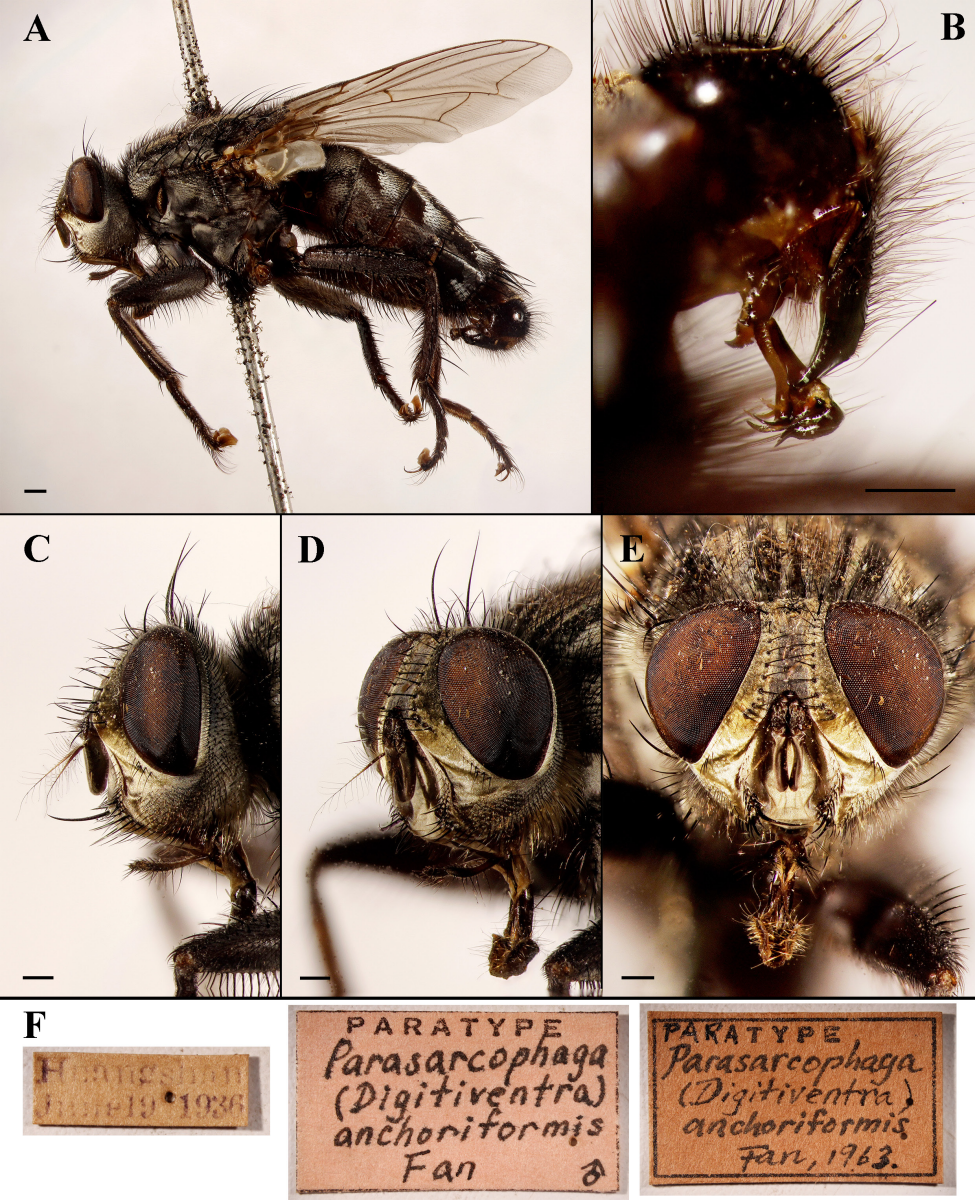
Paratype of Parasarcophaga (Digitiventra) anchoriformis Fan, 1964, male. **A**. Body, lateral view; **B**. Terminalia, lateral view; **C**. Head, lateral view; **D**. Head, anterolateral view; **E**. Head, anterior view; **F**. Labels. Scale bars: 1 mm.

**Name**. Parasarcophaga (Digitiventra) anchoriformis Fan, 1964: 309.

**Type locality**. China: Zhejiang, Mt Tianmushan.

**Material examined**. Holotype (♂): Zhejiang, Mt Tianmushan, 20.vii.1936, no further data. Paratype: 1 male, Anhui, Mt Huangshan, 19.iv.1936, no further data.

**Identity**. Sarcophaga (Robineauella) anchoriformis (Fan, 1964).

**References**. Parasarcophaga (Digitiventra) anchoriformis: Fan (1965: 278, 279), Chao and Fan (1993: 612), Meng (2003: 138).

*Robineauella anchoriformis*: Fan and Pape (1996: 255), Lei and Zhou (1998: 256), Fang and Wu (2001: 144).

Robineauella (Digitiventra) anchoriformis: Fan (1992: 695, 696), Zhang and Zhao (1992: 1170), Xue and Chao (1996: 1640, 1642), Xue and Tong (2001: 475), Meng (2003: 138), Verves (2020: 51).

Sarcophaga (Robineauella) anchoriformis: Pape (1996: 383), Wang (2013: 36), Zhang (2014: 21, 55).

**Remarks**. The type series is composed of the male holotype and nine paratypes. Fan (1964) did not label the paratypes, and the paratype label was attached later. Only the holotype and a single paratype were recovered in the present study.

**4. apiciscissa**

Fig. 5

**Figure 5.**
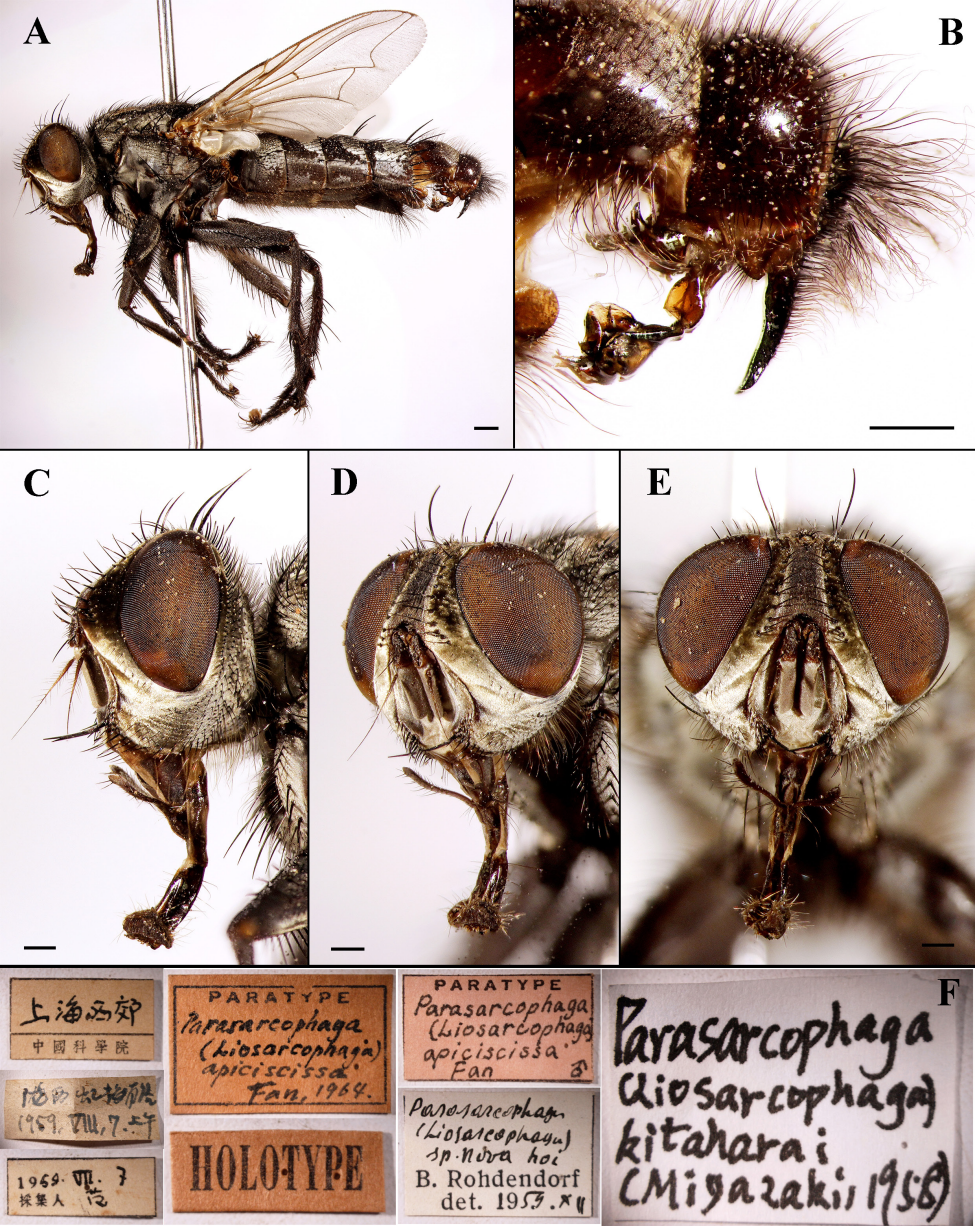
Holotype of Parasarcophaga (Liosarcophaga) apiciscissa Fan, 1964, male. **A**. Body, lateral view; **B**. Terminalia, lateral view; **C**. Head, lateral view; **D**. Head, anterolateral view; **E**. Head, anterior view; **F**. Labels. Scale bars: 1 mm.

**Name**. Parasarcophaga (Liosarcophaga) apiciscissa Fan, 1964: 310.

**Type locality**. China: Shanghai.

**Material examined**. Holotype (♂): Shanghai, Xijiao, 7.viii.1959, Zide Fan leg.

**Identity**. Sarcophaga (Liosarcophaga) kitaharai Miyazaki, 1958.

**References**. Parasarcophaga (Liosarcophaga) apiciscissa: Fan (1965: 284, 286.

Parasarcophaga (Liosarcophaga) kitaharai: Fan (1992: 713, 714), Xue and Chao (1996: 1609, 1615, 1618).

*Liosarcophaga kitaharai*: Fan and Pape (1996: 252), Verves (2020: 45).

Sarcophaga (Liosarcophaga) kitaharai: Pape (1996: 354), Wang (2013: 33); Zhang (2014: 17, 43).

**Remarks**. The type series is composed of the holotype and two paratypes, all males, but only the holotype was recovered in the present study.

**5. *bihami***

Fig. 6

**Figure 6.**
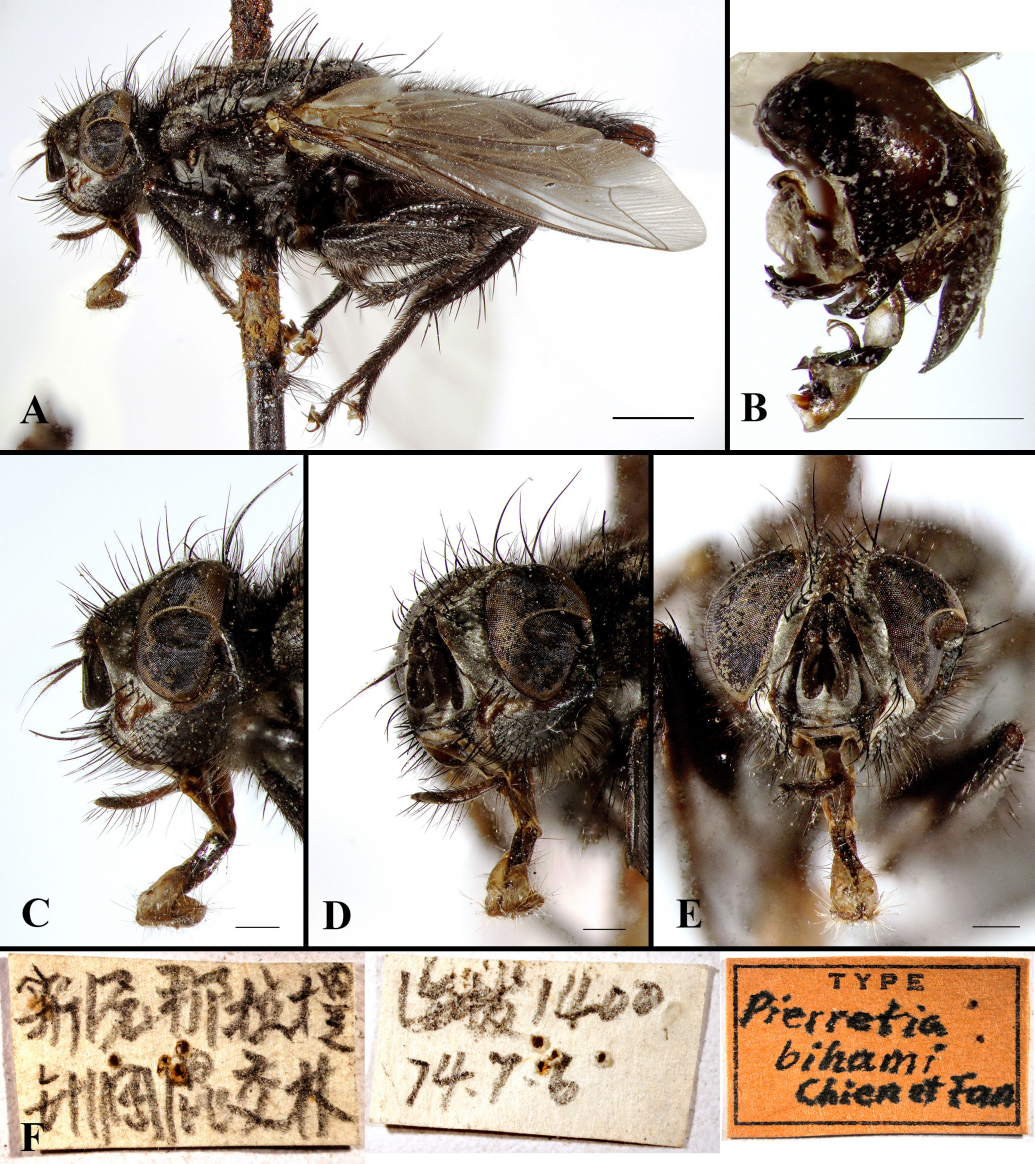
Type of *Pierretia bihami* Qian & Fan, 1981, male. **A**. Body, lateral view; **B**. Terminalia, lateral view; **C**. Head, lateral view; **D**. Head, anterolateral view; **E**. Head, anterior view; **F**. Labels. Scale bars: 1 mm.

**Name**. *Pierretia bihami* Qian & Fan, 1981: 446.

**Type locality**. China: Xinjiang, Xinyuan, Narathi.

**Material examined**. Holotype (♂): Xinjiang, Xinyuan, 6.vii.1974, Jinquan Qian leg.

**Identity**. Sarcophaga (Asceloctella) bihami (Qian & Fan, 1981).

**References**. *Asceloctella bihami*: Fan and Pape (1996: 247).

Athyrsomima (Sinopierretia) bihami: Verves (2020: 32).

*Pierretia bihami*: Fan (1992: 675, 676), Xue and Chao (1996: 1626, 1627, 1630), Xue and Wang (2006: 233).

Sarcophaga (Asceloctella) bihami: Pape (1996: 297), Wang (2013: 27), Zhang (2014: 10, 24).

**Remarks**. The type series is composed of a single specimen, the holotype by original designation. Qian and Fan (1981) mentioned that most abdominal spots are black and that the vesica is shaped like a pair of sclerotized hooks. The juxtal extension is developed and bifurcated for several parts, which can easily be recognized as lateral styli. The holotype is in good condition, but with the terminalia separated and glued to a small piece of cardboard (Fig. 6B). The label shows that this specimen is collected in mixed coniferous and broad-leaf deciduous forest at an altitude of 1400 m (Fig. 6F).

**6. chianshanensis**

Figs 7, 8

**Figure 7.**
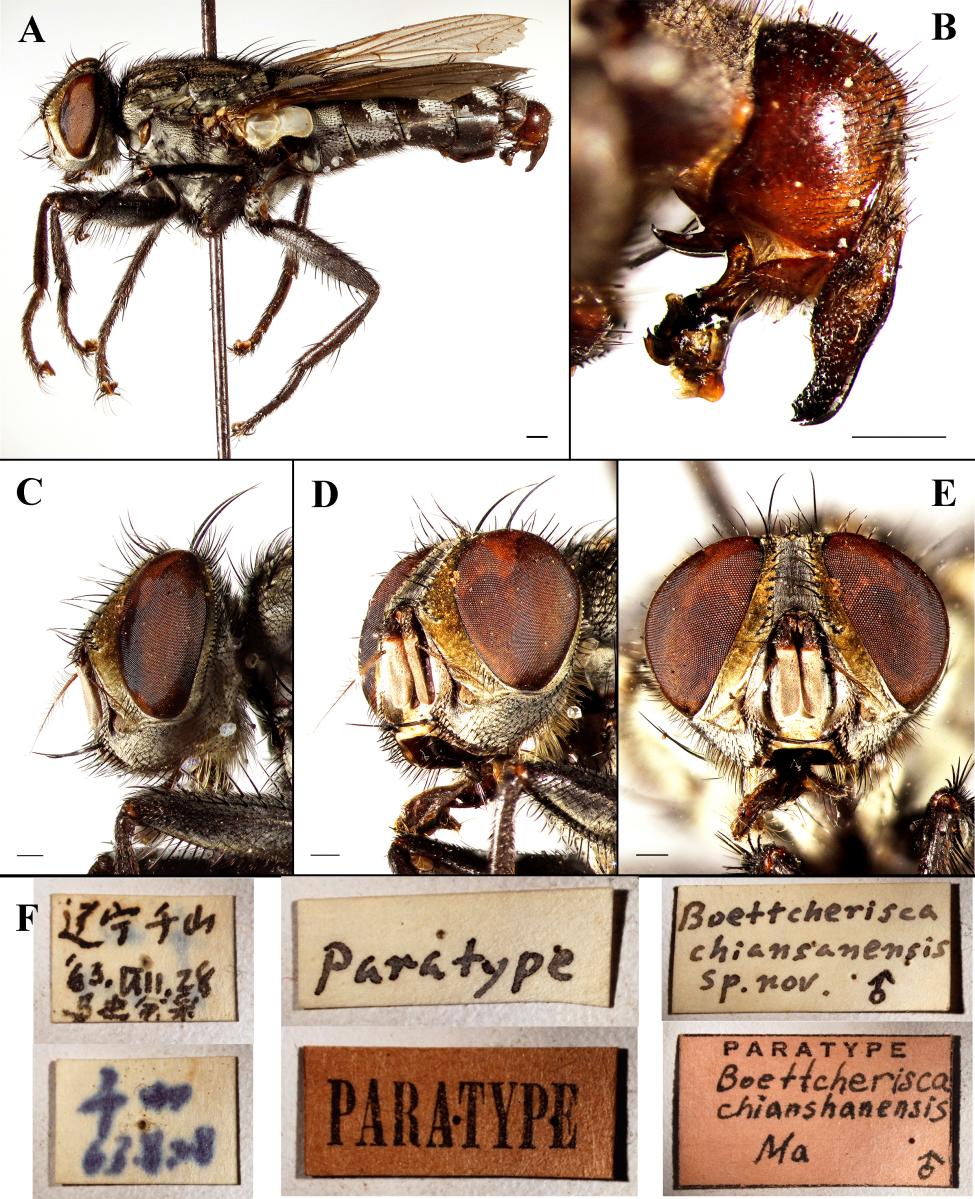
Paratype of *Boettcherisca chianshanensis* Ma, 1964, male. **A**. Body, lateral view; **B**. Terminalia, lateral view; **C**. Head, lateral view; **D**. Head, anterolateral view; **E**. Head, anterior view; **F**. Labels. Scale bars: 1 mm.

**Figure 8.**
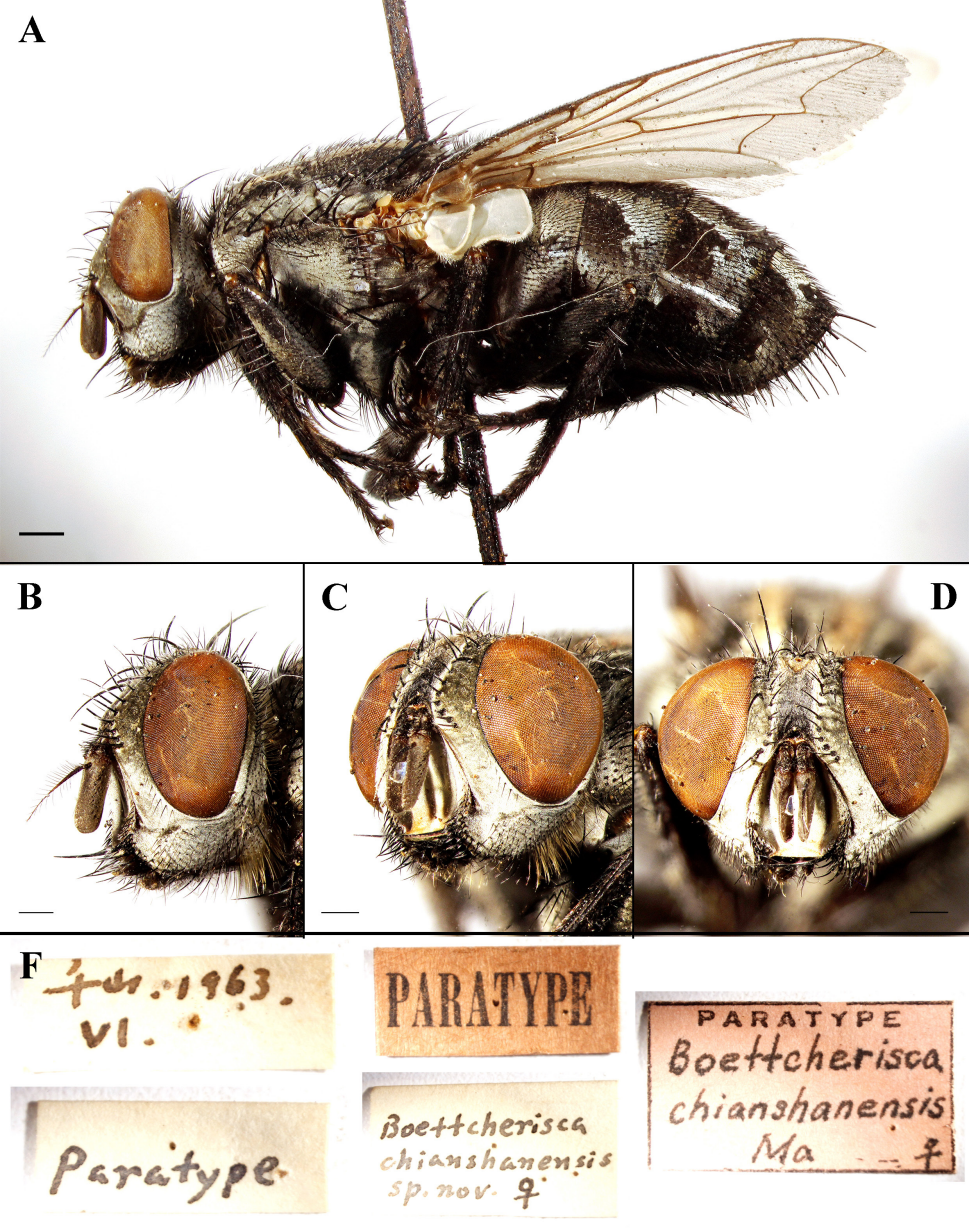
Paratype of *Boettcherisca chianshanensis* Ma, 1964, female. **A**. Body, lateral view; **B**. Head, lateral view; **C**. Head, anterolateral view; **D**. Head, anterior view; **F**. Labels. Scale bars: 1 mm.

**Name**. *Boettcherisca chianshanensis* Ma, 1964: 59.

**Type locality**. China: Liaoning, Chianshan.

**Material examined**. Paratypes: 1♂, Liaoning, Qianshan (Chianshan), 28.viii.1963; no further data; 1♀, Liaoning, Qianshan (Chianshan), vi.1963; no further data.

**Identity**. Sarcophaga (Boettcherisca) nathani (Lopes, 1961).

**References**. *Boettcherisca nathani*: Fan and Pape (1996: 248), Verves (2020: 56).

Sarcophaga (Boettcherisca) nathani: Pape (1996: 310), Zhang (2014: 12, 28).

**Remarks**. The type series is composed of the male holotype, one female labelled as allotype, plus 33 male and 21 female paratypes. The holotype was stated to be deposited in Shenyang Medical College. Only a male and a female paratype were recovered in the present study.

**7. *gracilior***

Fig. 9

**Figure 9.**
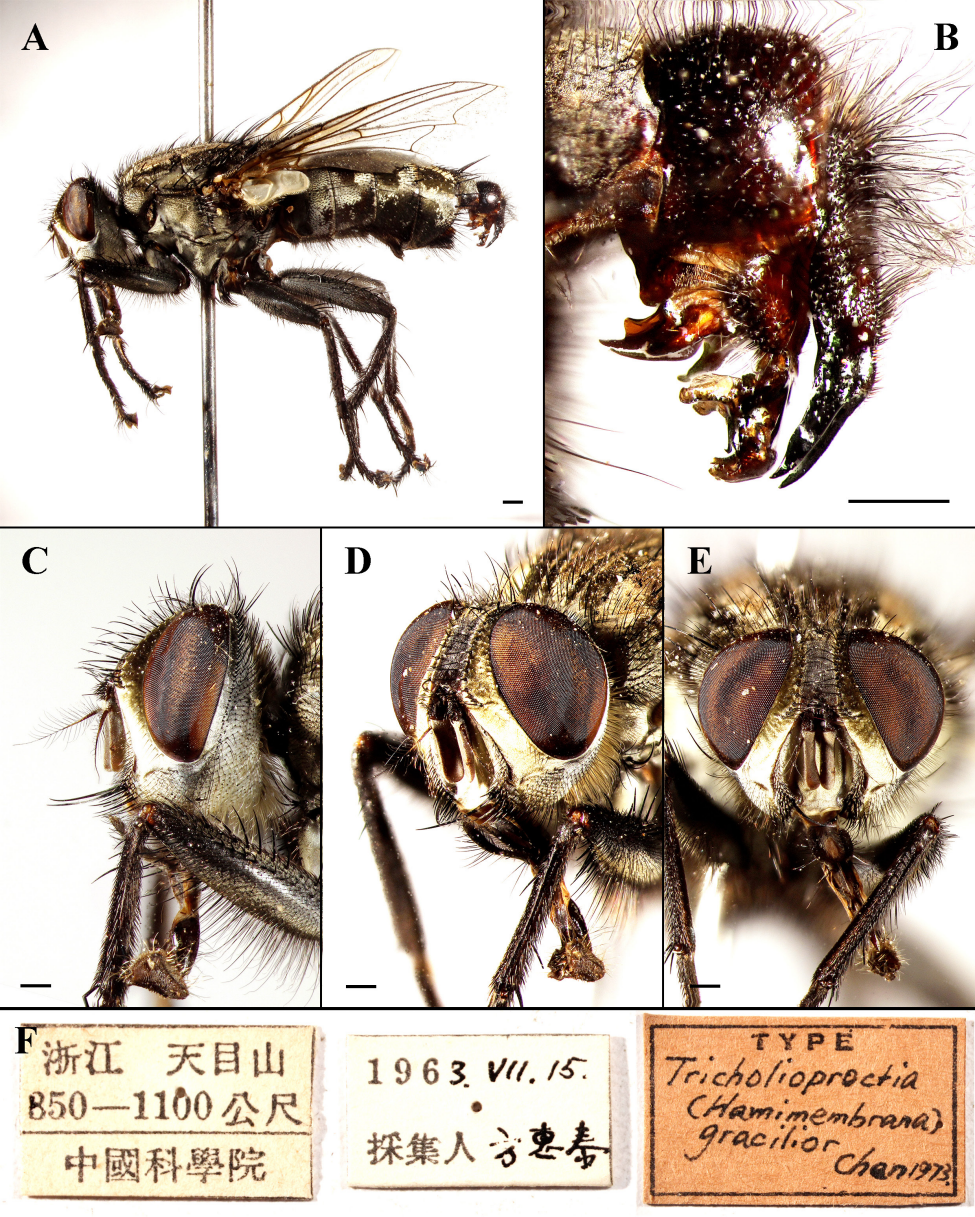
Holotype of Tricholioproctia (Hamimembrana) gracilior Chen, 1975, male. **A**. Body, lateral view; **B**. Terminalia, lateral view; **C**. Head, lateral view; **D**. Head, anterolateral view; **E**. Head, anterior view; **F**. Labels. Scale bars: 1 mm.

**Name**. Tricholioproctia (Hamimembrana) gracilior Chen, 1975: 115.

**Type locality**. China: Zhejiang, Mt Tianmushan.

**Material examined**. Holotype (♂): Zhejiang, Mt Tianmushan, 850–1100 m, 15.vii.1965, Huitai Fang leg. Paratypes: 4 males, same label data as holotype.

**Identity**. Sarcophaga (Sarcorohdendorfia) gracilior (Chen, 1975).

**References**. Tricholioproctia (Hamimembrana) gracilior: Fang and Wu (2001: 144), Wei and Yang (2007: 532).

*Sarcorohdendorfia gracilior*: Fan (1992: 659, 662), Xue and Chao (1996: 1646, 1652), Fan and Pape (1996: 256), Xue and Tong (2001: 476), Wei (2005: 406), Chen et al. (2010: 281), Yue (2016: 32, 87), Verves (2020: 53).

Sarcophaga (Sarcorohdendorfia) gracilior: Pape (1996: 397), Wang (2013: 37), Zhang (2014: 22, 58).

**Remarks**. The type series is composed of the holotype plus seven paratypes, all males with identical data. The holotype and four paratypes were recovered in this study.

**8. *hinglungensis***

Fig. 10

**Figure 10.**
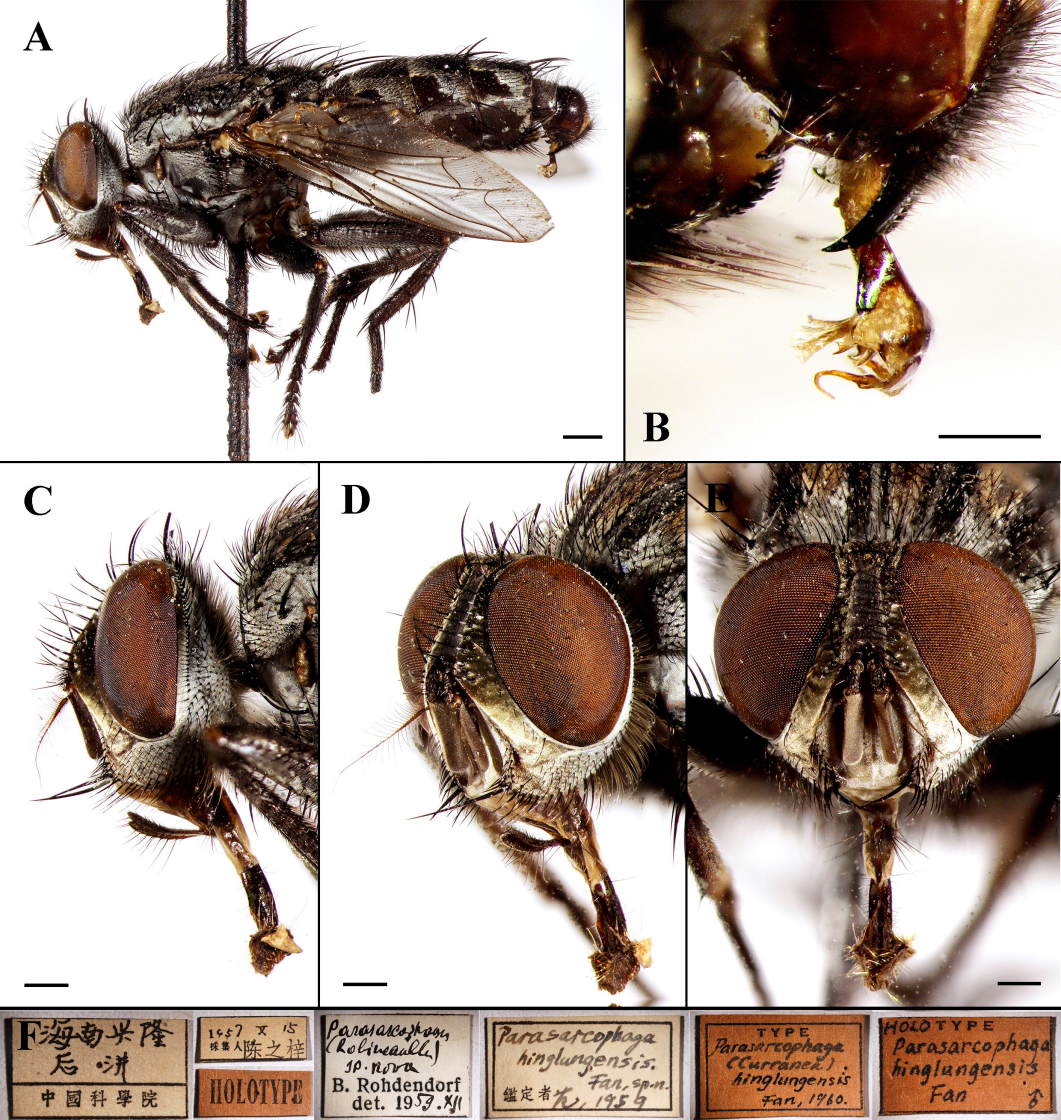
Holotype of Parasarcophaga (Curranea) hinglungensis Fan, 1964, male. **A**. Body, lateral view; **B**. Terminalia, lateral view; **C**. Head, lateral view; **D**. Head, anterolateral view; **E**. Head, anterior view; **F**. Labels. Scale bars: 1 mm.

**Name**. Parasarcophaga (Curranea) hinglungensis Fan, 1964: 307.

**Type locality**. China: Hainan, Hinglung.

**Material examined**. Holotype (♂): Hainan, Hinglung, 15.x.1975, Zhizi Chen leg.

**Identity**. Sarcophaga (Pandelleisca) hinglungensis (Fan, 1964).

**References**. Liosarcophaga (Curranea) hinglungensis: Fan and Pape (1996: 252).

*Liosarcophaga* (s. str.) *hinglungensis*: Verves (2020: 44).

Parasarcophaga (Curranea) hinglungensis: Fan (1965: 276, 277), Wang et al. (1992: 171), Fan (1992: 708, 709), Xue and Chao (1996: 1609, 1614, 1620), Lei and Zhou (1998: 255), Fang and Wu (2001: 144), Xue and Tong (2001: 474), Xue and Song (2002: 810, 812), Liu et al. (2008: 72), Yue (2016: 62, 130).

Sarcophaga (Liosarcophaga) hinglungensis: Pape (1996: 352), Wang (2013: 32), Zhang (2014: 16, 42).

**Remarks**. The type series is composed of one holotype, 9 male syntypes from Hainan, and a single male syntype from Hubei province. Only the holotype was recovered in this study.

**9. *huangshanensis***

Figs 11, 12

**Figure 11.**
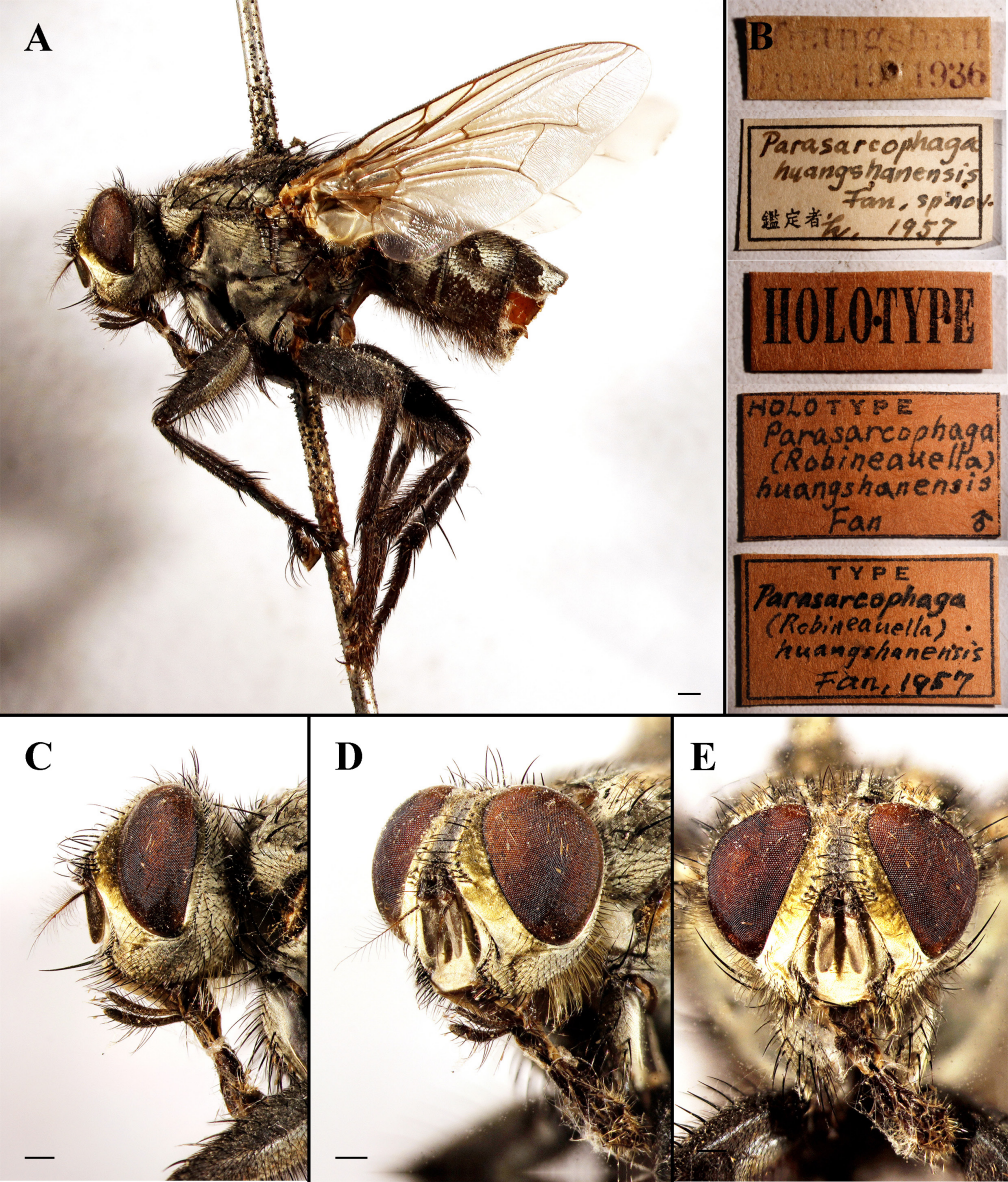
Holotype of Parasarcophaga (Robineauella) huangshanensis Fan, 1964, male. **A**. Body, lateral view; **B**. Labels; **C**. Head, lateral view; **D**. Head, anterolateral view; **E**. Head, anterior view. Scale bars: 1 mm.

**Figure 12.**
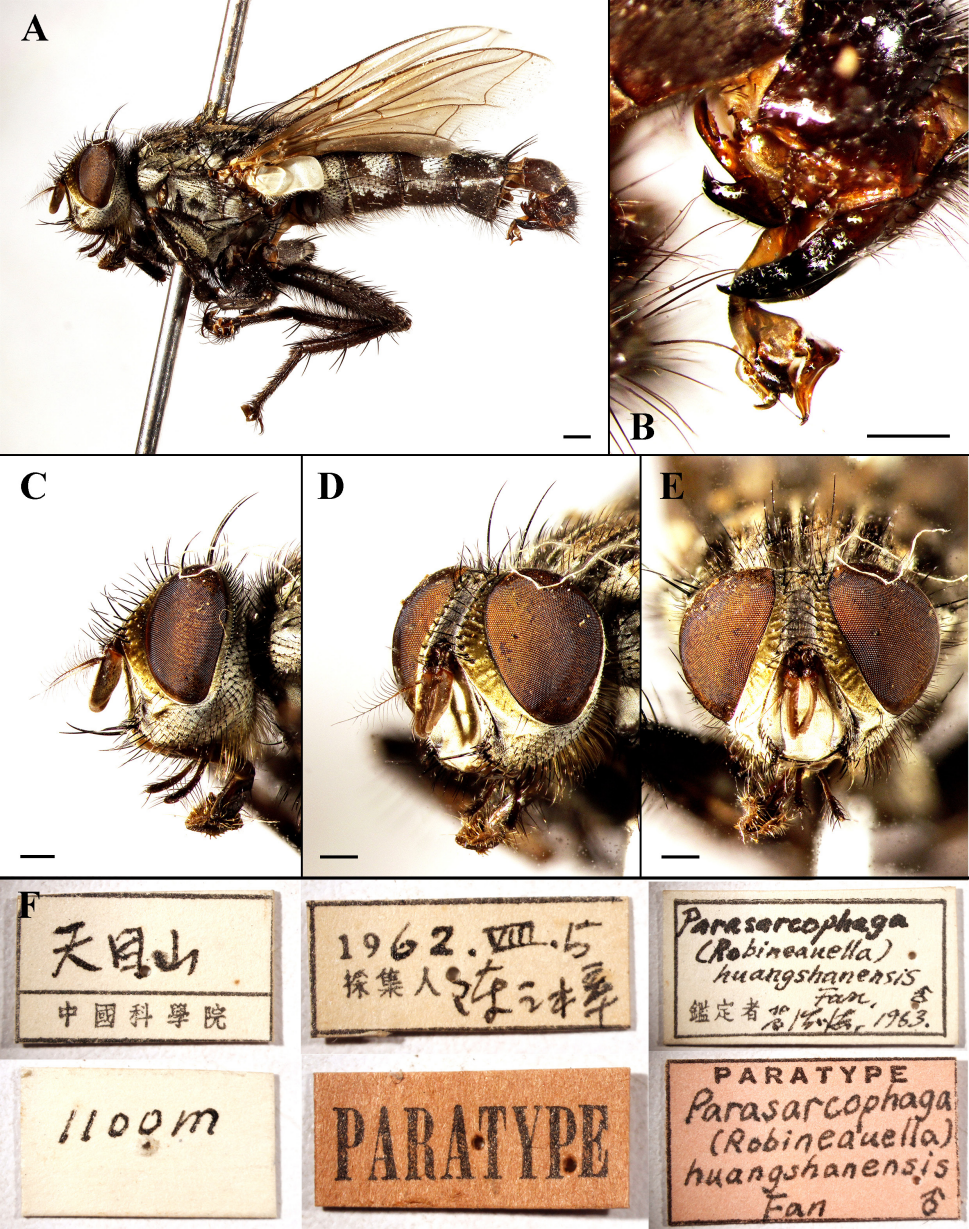
Paratype of Parasarcophaga (Robineauella) huangshanensis Fan, 1964, male. **A**. Body, lateral view; **B**. Terminalia, lateral view; **C**. Head, lateral view; **D**. Head, anterolateral view; **E**. Head, anterior view; **F**. Labels. Scale bars: 1 mm.

**Name**. Parasarcophaga (Robineauella) huangshanensis Fan, 1964: 312.

**Type locality**. China: Anhui, Huang-Shan.

**Material examined**. Holotype (♂): Anhui, Mt Huangshan, 19.vi.1936, no further data.

Paratypes: 2 males, Zhejiang, Mt Tianmushan, 1100 m, 5.vii.1962, Zhizi Chen leg.

**Identity**. Sarcophaga (Robineauella) huangshanensis (Fan, 1964).

**References**. Parasarcophaga (Robineauella) huangshanensis: Fan (1965: 281, 282), Chao and Fan (1993: 613).

*Robineauella huangshanensis*: Fan and Pape (1996: 255), Lei and Zhou (1998: 256), Fang and Wu (2001: 144), Meng (2003: 138), Ma and Wu (2010: 265).

*Robineauella* (s. str.) *huangshanensis*: Fan (1992: 696, 697), Xue and Chao (1996: 1640, 1641, 1642), Xue and Tong (2001: 475), Zhang (2005: 846), Verves (2020: 52).

Sarcophaga (Robineauella) huangshanensis: Pape (1996: 384), Wang (2013: 36), Zhang (2014: 21, 55).

**Remarks**. The type series is composed of the holotype and two paratypes, all males, and all of which were recovered in the present study.

**10. *lhasae***

Figs 13, 14

**Figure 13.**
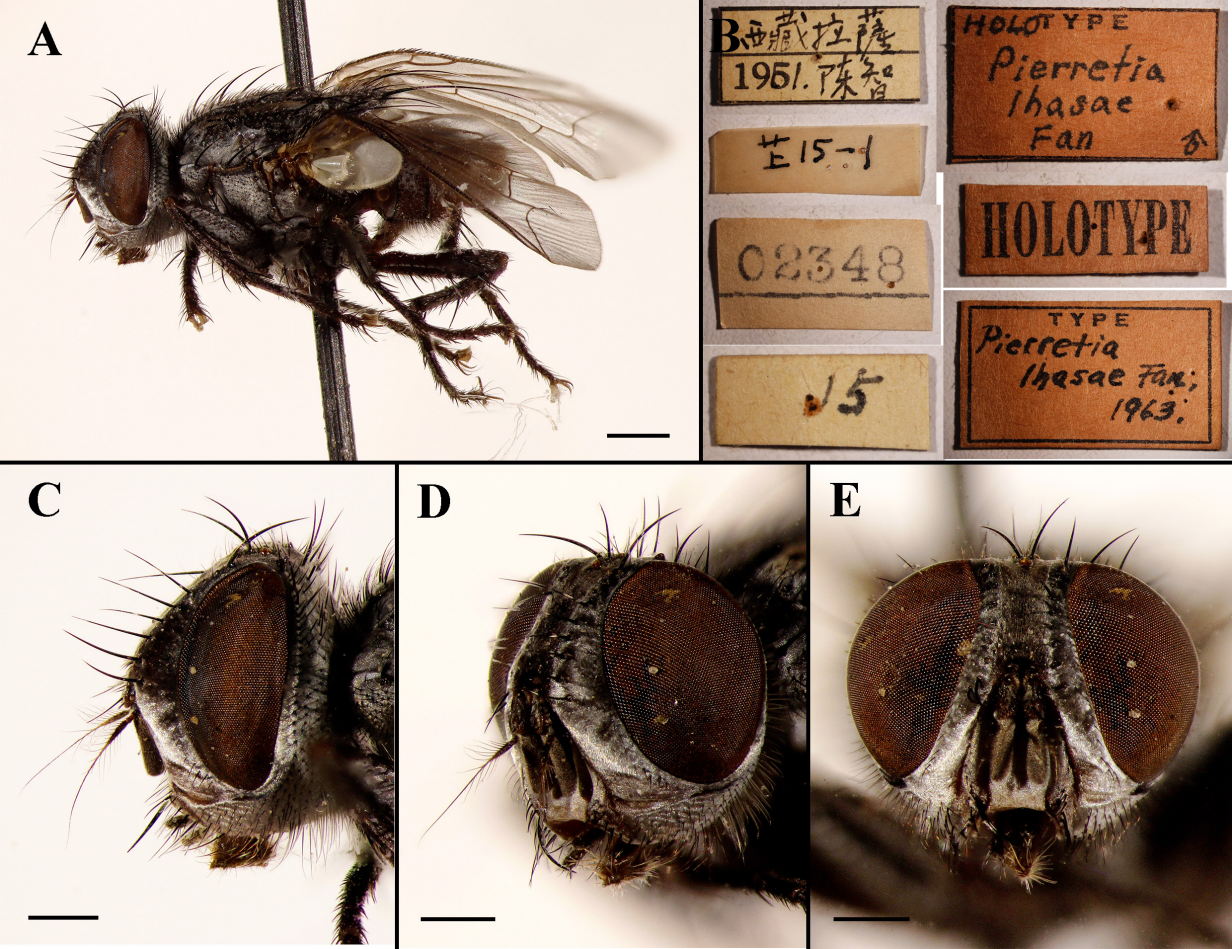
Holotype of Pierretia (Pseudothyrsocnema) lhasae Fan, 1964, male. **A**. Body, lateral view; **B**. Labels; **C**. Head, lateral view; **D**. Head, anterolateral view; **E**. Head, anterior view. Scale bars: 1 mm.

**Figure 14.**
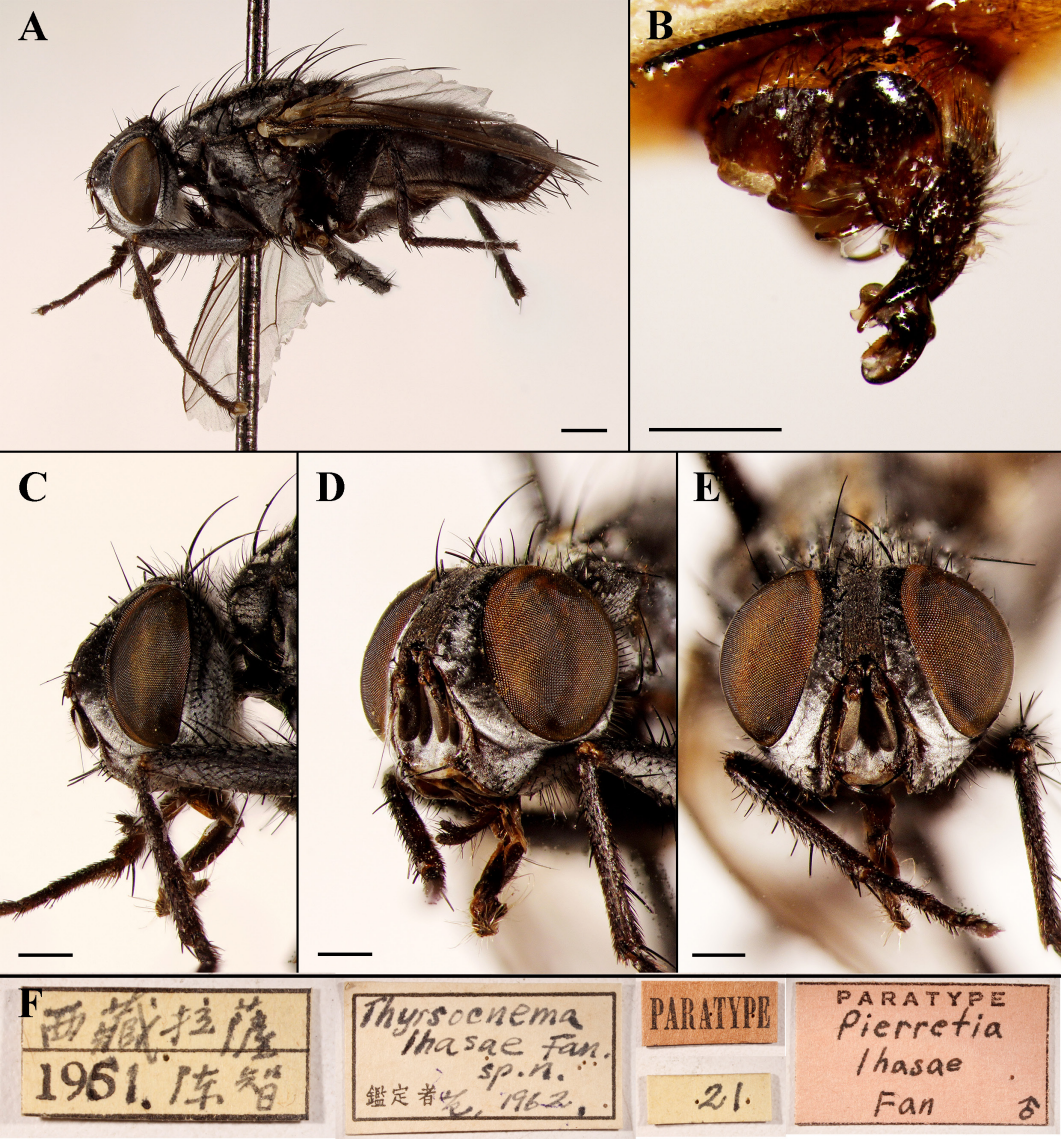
Paratype of Pierretia (Pseudothyrsocnema) lhasae Fan, 1964, male. **A**. Body, lateral view; **B**. Terminalia, lateral view; **C**. Head, lateral view; **D**. Head, anterolateral view; **E**. Head, anterior view; **F**. Labels. Scale bars: 1 mm.

**Name**. Pierretia (Pseudothyrsocnema) lhasae Fan, 1964: 306.

**Type locality**. China: Xizang, Lhasa.

**Material examined**. Holotype (♂): Xizang, Lhasa, 1961, Zhi Chen leg., no further data. Paratype: 1 male, same label data as holotype.

**Identity**. Sarcophaga (Pseudothyrsocnema) lhasae (Fan, 1964).

**References**. Pierretia (Pseudothyrsocnema) lhasae: Fan (1965: 236, 237), Fan (1992: 685, 686), Xue and Chao (1996: 1630, 1634, 1635), Xue and Wang (2006: 235), Yue (2016: 47, 110).

*Pseudothyrsocnema lhasae*: Chao and Zhang (1982: 230; as *Pierretia lhasae*); Fan and Pape (1996: 255), Verves (2020: 38).

Sarcophaga (Pseudothyrsocnema) lhasae: Pape (1996: 382), Wang (2013: 35), Zhang (2014: 20, 53).

**Remarks**. The type series is composed of the holotype and six paratypes, all males. Only the holotype and a single paratype were recovered in the present study.

**11. *liukiuensis***

Fig. 15

**Figure 15.**
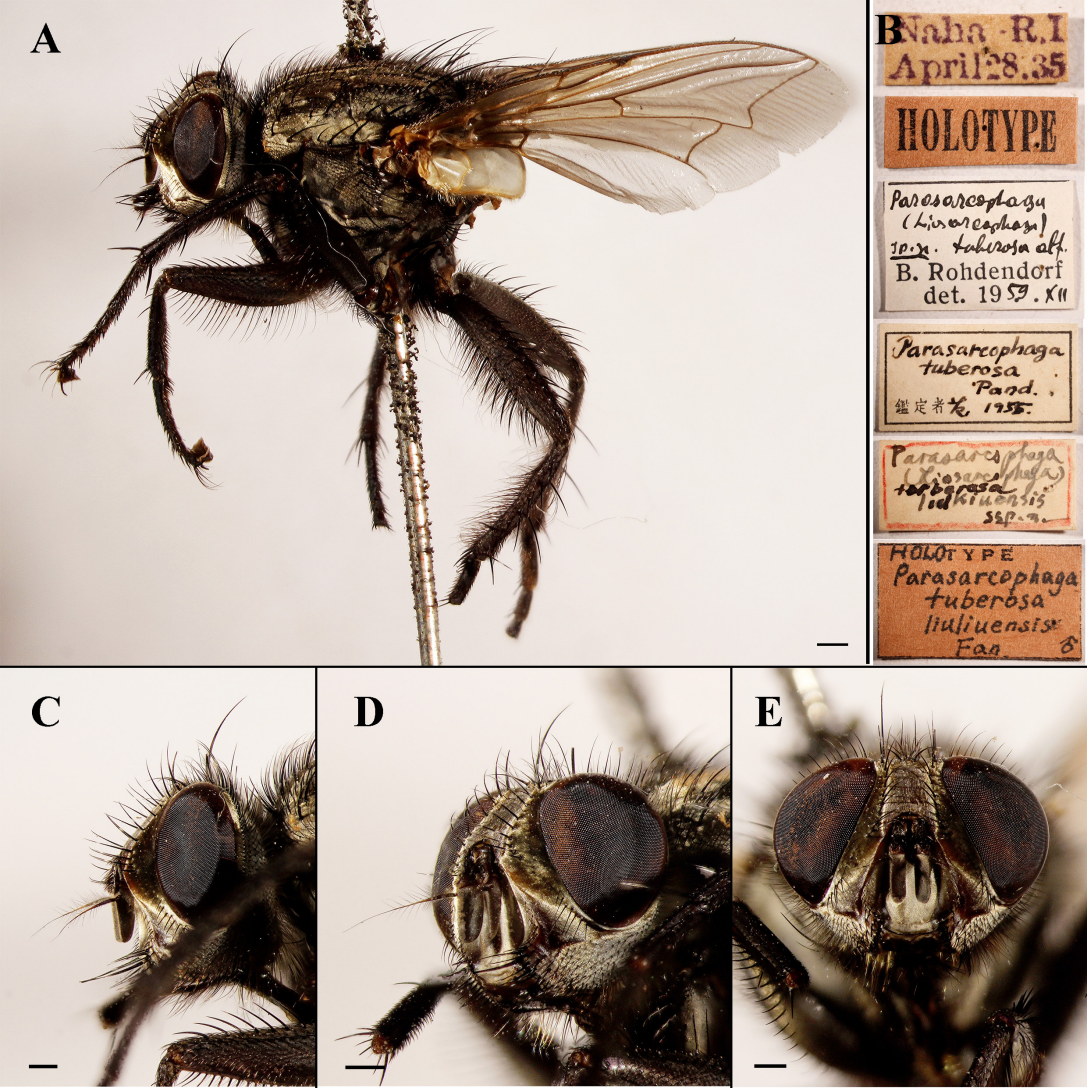
Holotype of Parasarcophaga (Liosarcophaga) tuberosa liukiuensis Fan, 1964, male. **A**. Body, lateral view; **B**. Labels; **C**. Head, lateral view; **D**. Head, anterolateral view; **E**. Head, anterior view. Scale bars: 1 mm.

**Name**. Parasarcophaga (Liosarcophaga) tuberosa liukiuensis Fan, 1964: 311.

**Type locality**. Japan, Ryukyu Is, Naha.

**Material examined**. Holotype (♂): Japan, Ryukyu Is, Naha, 28.iv.1935, no further data.

**Identity**. Sarcophaga (Liosarcophaga) liukiuensis Fan, 1964.

**References**. Parasarcophaga (Liosarcophaga) tuberosa: Fan (1965: 267, 287, 288), Fan (1992: 714, 715), Zhang and Zhao (1992: 1171), Xue and Chao (1996: 1609, 1620, 1622), Zhang and Chao (1996: 246), Xue and Wang (2006: 233), Liu et al. (2008: 72), Yue (2016: 66, 137).

*Parasarcophaga tuberosa*: Chao and Zhang (1985: 132), Wang et al. (1992: 171), Shi (1993: 512), Yu and Sun (1993: 423), Zhang et al. (1994: 282), Lei and Zhou (1998: 255).

*Liosarcophaga liukiuensis*: Fan and Pape (1996: 252), Verves (2020: 46).

*Liosarcophaga tuberosa*: Fan and Pape (1996: 253), Verves (2020: 47).

Sarcophaga (Liosarcophaga) liukiuensis: Pape (1996: 354), Zhang (2014: 17, 43).

Sarcophaga (Liosarcophaga) tuberosa: Wang (2013: 33), Zhang (2014: 17, 44).

Parasarcophaga (Liosarcophaga) tuberosa liukiuensis: Fan (1965: 287, 288), Chao and Fan (1993: 612).

*Sarcophaga tuberosa*: Hu (1940: 427).

**Remarks**. The type series is composed of a single specimen, the male holotype by original designation.

**12. *nigribasicosta***

Fig. 16

**Figure 16.**
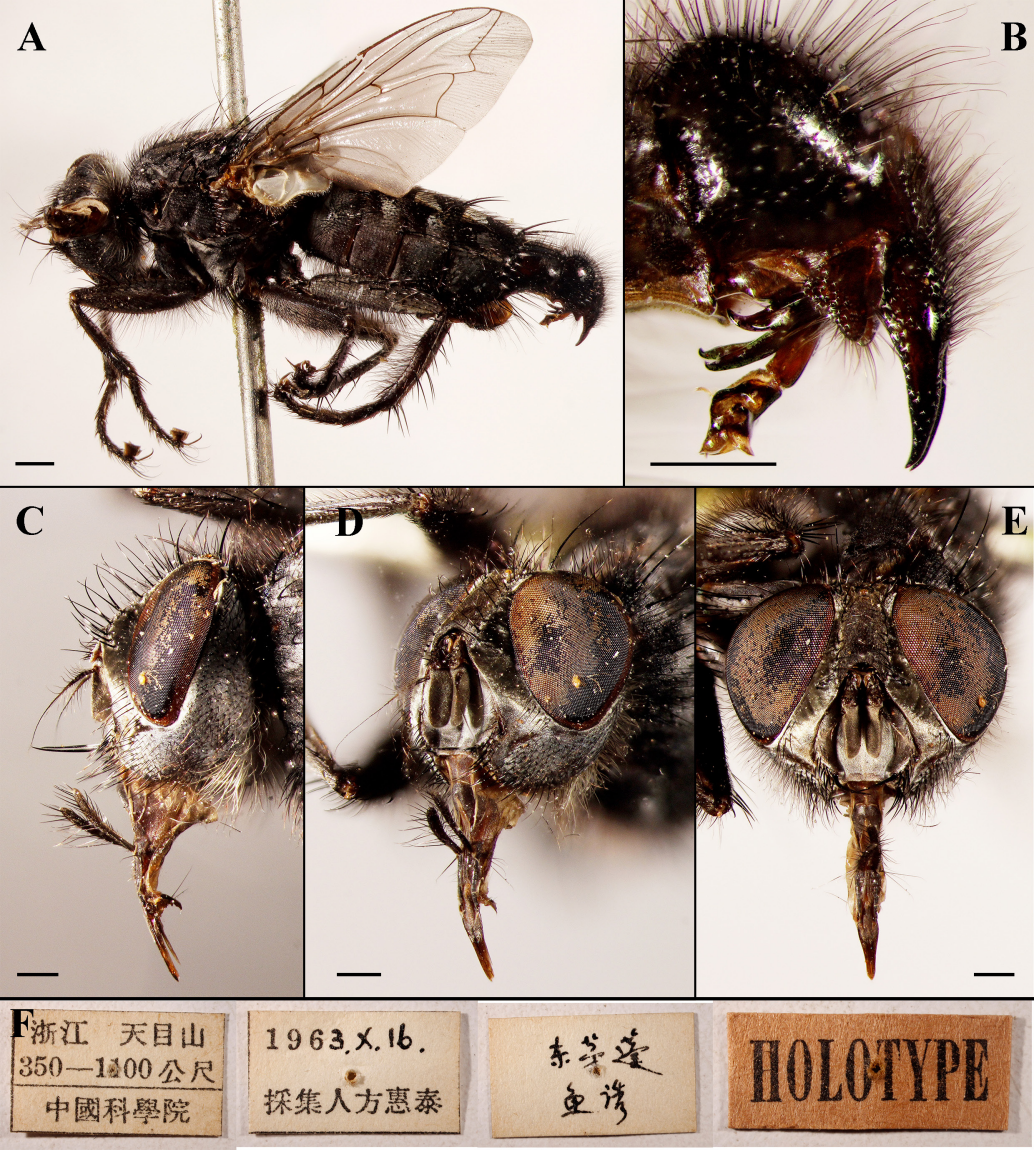
Holotype of *Dinemomyia nigribasicosta* Chen, 1975, male. **A**. Body, lateral view; **B**. Terminalia, lateral view; **C**. Head, lateral view; **D**. Head, anterolateral view; **E**. Head, anterior view; **F**. Labels. Scale bars: 1 mm.

**Name**. *Dinemomyia nigribasicosta* Chen, 1975: 114.

**Type locality**. China, Zhejiang, Tianmu-Shan.

**Material examined**. Holotype (♂): Zhejiang, Tianmu Mountain (Tianmu-Shan), 350–1100 m, 6.x.1963, Huitai Fang leg. Paratypes: 2 males, Zhejiang, Tianmu mountain (Tianmu-Shan), 1500 m, 12.ix.1963, Huitai Fang leg.

**Identity**. Sarcophaga (Dinemomyia) nigribasicosta (Chen, 1975).

**References**. *Dinemomyia nigribasicosta*: Fan (1992: 651), Xue and Chao (1996: 1587, 1588), Fan and Pape (1996: 249), Fang and Wu (2001: 143), Xue and Tong (2001: 472), Ma and Wu (2010: 265), Verves (2020: 34).

Sarcophaga (Dinemomyia) nigribasicosta: Pape (1996: 314), Wang (2013: 29), Zhang (2014: 12, 29).

**Remarks**. The entire type series is composed of the holotype and two paratypes, all males, all of which were recovered in the present study.

**13. *otiophalla***

Fig. 17

**Figure 17.**
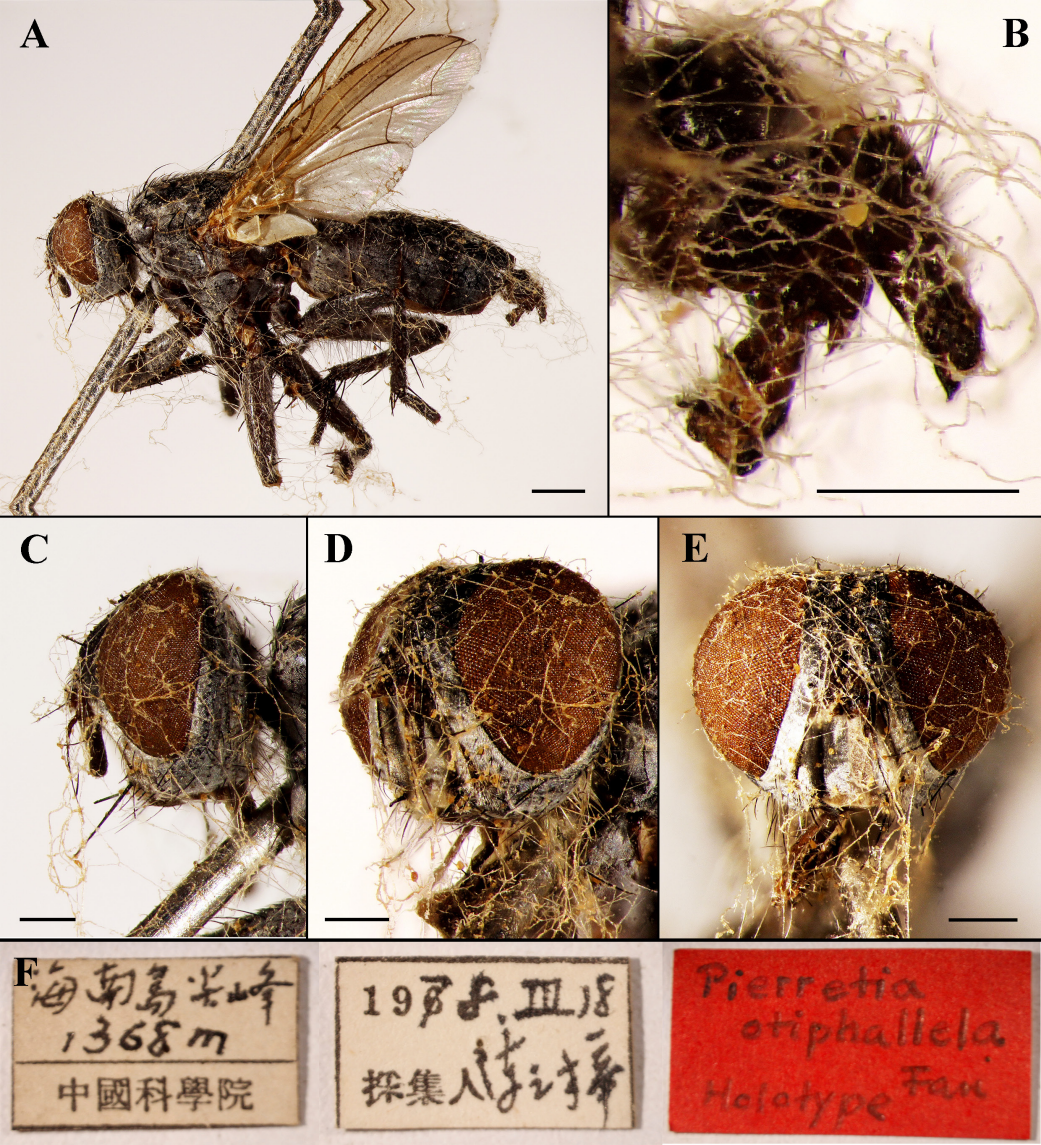
Holotype of Pierretia (Mehria) otiophalla Fan & Chen, 1981, male. **A**. Body, lateral view; **B**. Terminalia, lateral view; **C**. Head, lateral view; **D**. Head, anterolateral view; **E**. Head, anterior view; **F**. Labels. Scale bars: 1 mm.

**Name**. Pierretia (Mehria) otiophalla Fan & Chen, 1981: 241.

**Type locality**. China: Hainan, Mt Jianfeng.

**Material examined**. Holotype (♂): Hainan, Jianfengling (Mt Jianfeng), 1368 m, 18.iii.1978, Zhizi Chen leg.

**Identity**. Sarcophaga (Kalshovenella) otiophalla (Fan & Chen, 1981).

**References**. *Mehria otiophalla*: Fan and Pape (1996: 253).

Pierretia (Arachnidomyia) otiophalla: Fan (1992: 678, 680), Xue and Chao (1996: 1628, 1634, 1636), Xue and Song (2002: 809, 812).

Sarcophaga (Kalshovenella) otiophalla: Pape (1996: 340), Wang (2013: 30), Zhang (2014: 14, 37).

**Remarks**. The type series is composed of a single specimen, the holotype by original designation.

**14. *parva***

Figs 18, 19

**Figure 18.**
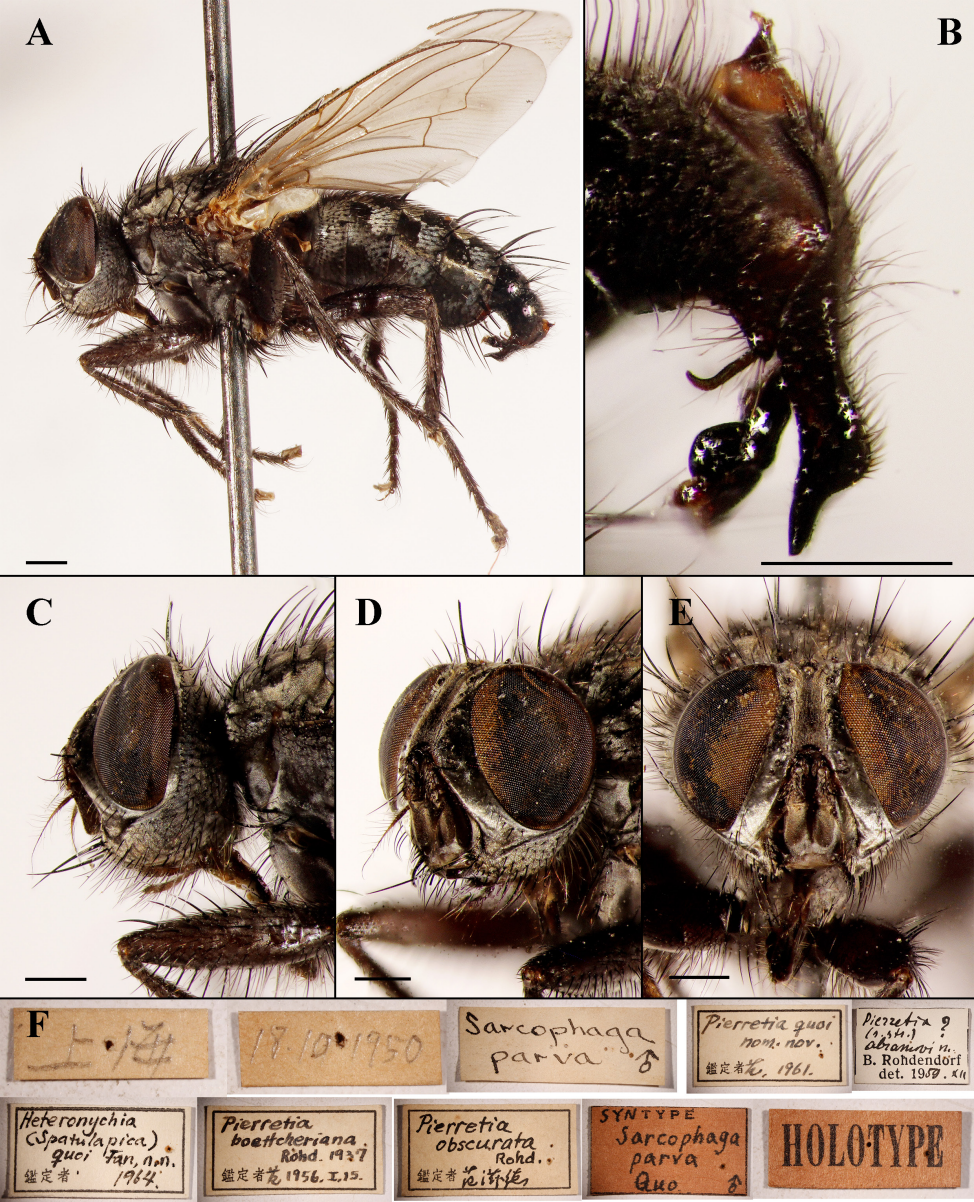
Holotype of *Sarcophaga parva* Quo, 1952, male. **A**. Body, lateral view; **B**. Labels; **C**. Head, lateral view; **D**. Head, anterolateral view; **E**. Head, anterior view. Scale bars: 1 mm.

**Figure 19.**
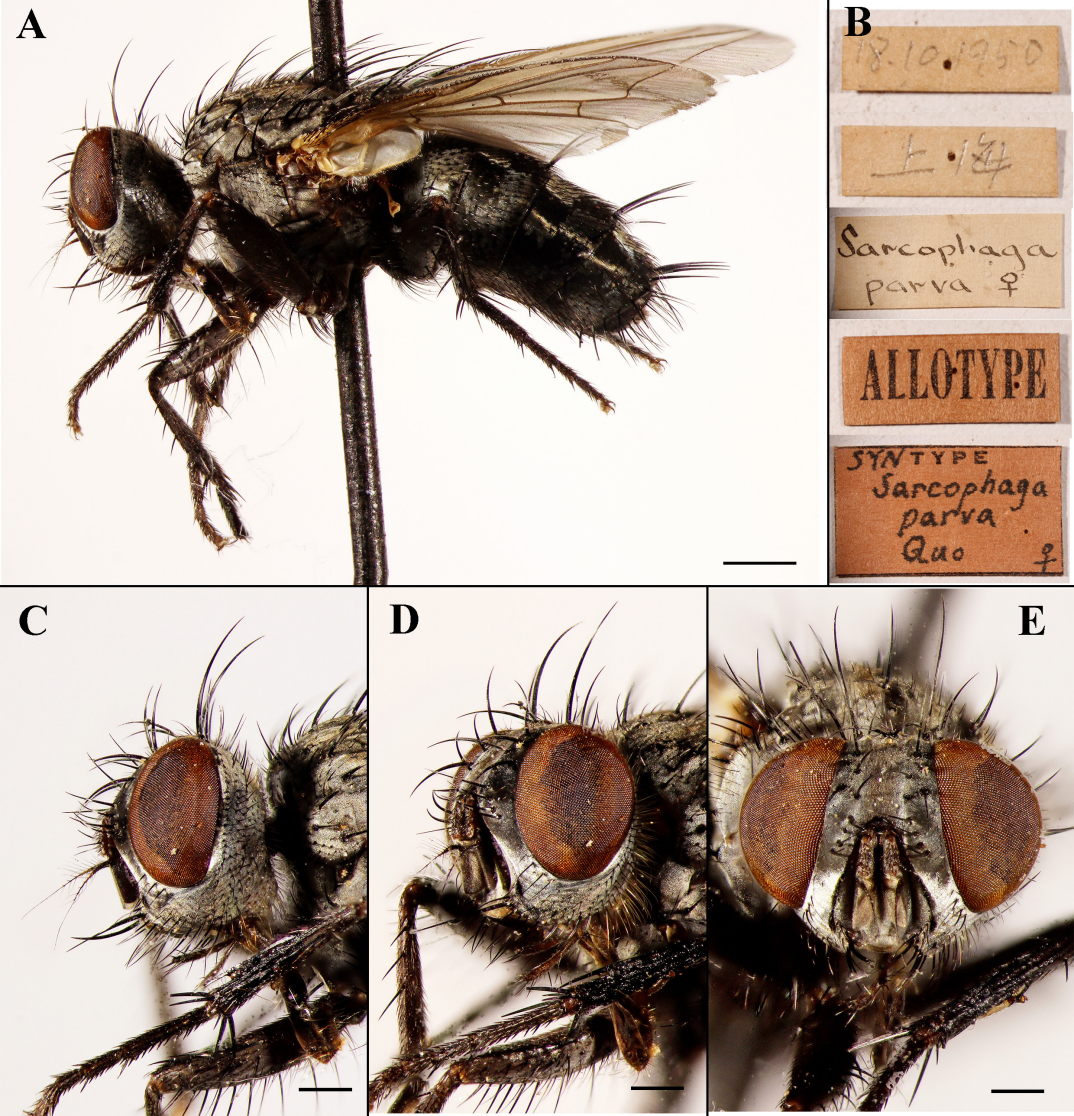
Allotype of *Sarcophaga parva* Quo, 1952, female. **A**. Body, lateral view; **B**. Labels; **C**. Head, lateral view; **D**. Head, anterolateral view; **E**. Head, anterior view. Scale bars: 1 mm.

**Name**. *Sarcophaga parva* Quo, 1952: 67.

**Type locality**. China: Shanghai.

**Material examined**. Syntypes (3♂♂, 1♀): 1 male (labelled holotype), Shanghai, 18.x.1950, no further data; 1 male, Guangxi, Guilin, 19.viii.1935, no further data; 1 male, Shanghai, 29.ix.1950, no further data; 1 female (labelled allotype), Shanghai, 18.x.1950, no further data.

**Identity**. Sarcophaga (Heteronychia) depressifrons Zetterstedt, 1845.

**References**. *Heteronychia* (s. str.) depressifrons: Verves (2020: 29).

*Heteronychia* (s. str.) quoi: Fan (1992: 652), Whitmore (2010: 59), Yue (2016: 29, 82).

*Heteronychia quoi*: Xue and Chao (1996: 1593, 1595, 1597), Fan and Pape (1996: 249), Lei and Zhou (1998: 254).

Sarcophaga (Heteronychia) depressifrons: Pape (1996: 325), Whitmore (2010: 59), Wang (2013: 15, 29), Zhang (2014: 13, 32).

**Remarks**. The type series is composed of six males and five females but only three males and one female were recovered in the present study. Quo (1952) did not designate a holotype, which means that the specimens are syntypes; the holotype and allotype labels are here considered later additions. *Sarcophaga parva* Quo, 1952 is a secondary junior homonym of *Pierretia parva* Robineau-Desvoidy, 1863, and Heteronychia (Spatulapica) quoi Fan, 1964 was proposed as a new replacement name.

**15. *pudongensis***

**Name**. *Beziella pudongensis* Fan, Chen & Lu, 2003: 80.

**Type locality**. China: Shanghai, Pudong, northern neighbourhood of Pudong International Airport.

**Material examined**. None.

**Identity**. Sarcophaga (Beziella) pudongensis (Fan, Chen & Lu, 2003).

**References**. *Sarcorohdendorfia pudongensis*: Verves (2020: 54).

**Remarks**. The type series is composed of the male holotype plus two male and one female paratypes, but a depository is given only for the holotype, which was stated to be deposited at the Shanghai Entomological Museum (“the holotype specimen is kept in Shanghai Institute of Entomology”). None of the original type specimens were recovered in the present study.

**16. *recurvata***

Fig. 20

**Figure 20.**
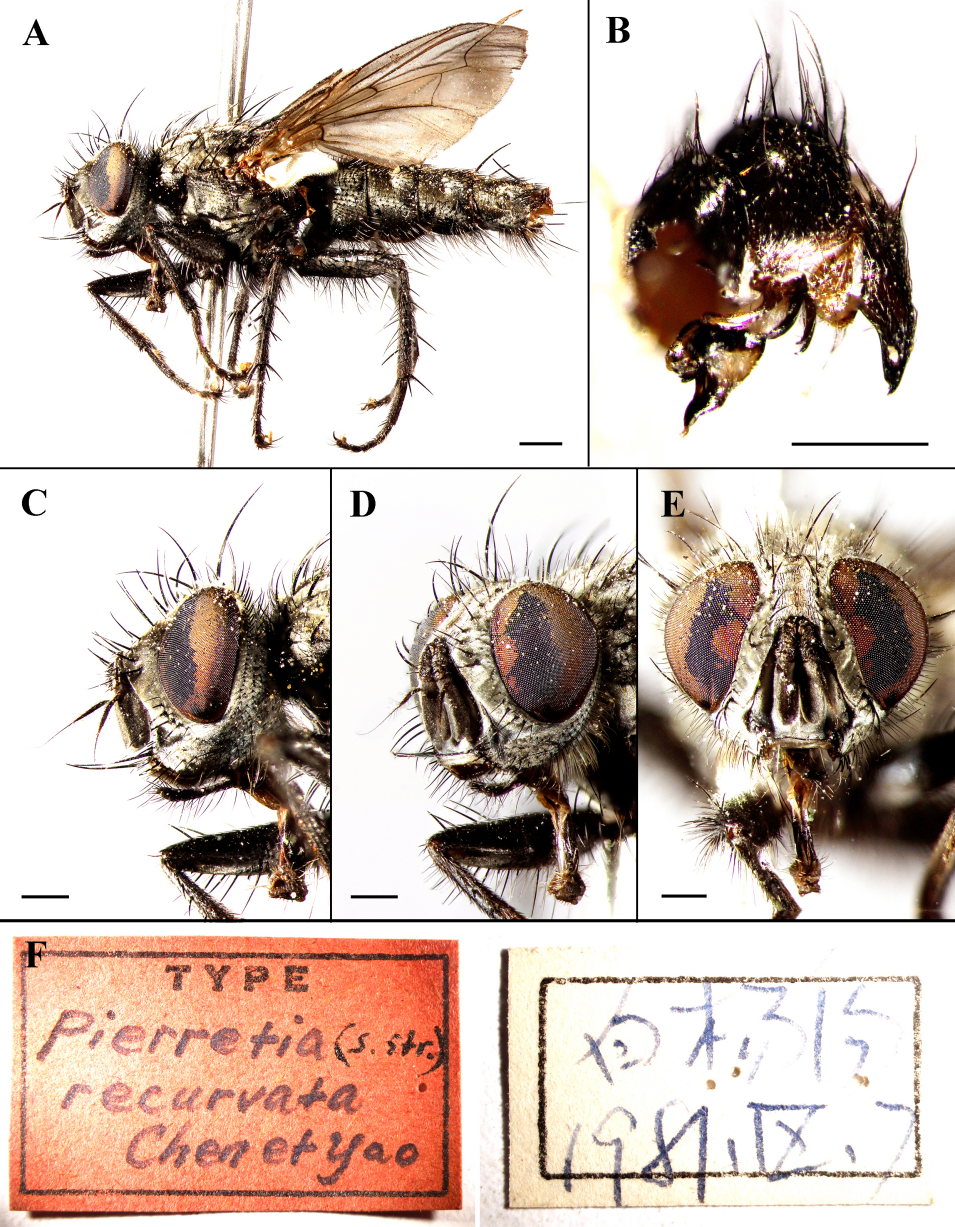
Holotype of *Pierretia* (s. str.) *recurvata* Chen & Yao, 1985, male. **A**. Body, lateral view; **B**. Terminalia, lateral view; **C**. Head, lateral view; **D**. Head, anterolateral view; **E**. Head, anterior view; **F**. Labels. Scale bars: 1 mm.

**Name**. *Pierretia* (s. str.) *recurvata* Chen & Yao, 1985: 295.

**Type locality**. China: Xinjiang. Ürümqi (Bai Yang Gou).

**Material examined**. Holotype (♂): Xinjiang, Ürümqi (Bai Yang Gou), 7.ix.1981, no further data.

**Identity**. Sarcophaga (Myorhina) recurvata (Chen & Yao, 1985).

**References**. *Mehria recurvata*: Fan and Pape (1996: 253).

*Myorhina* (s. str.) *recurvata*: Verves (2020: 35).

*Pierretia* (s. str.) *recurvata*: Fan (1992: 674, 675), Xue and Chao (1996: 1627, 1634, 1635).

Sarcophaga (Myorhina) recurvata: Pape (1996: 365), Wang (2013: 33), Zhang (2014: 18, 47).

**Remarks**. The type series is composed of only one holotype.

**17. shanghaiensis**

Figs 21, 22

**Figure 21.**
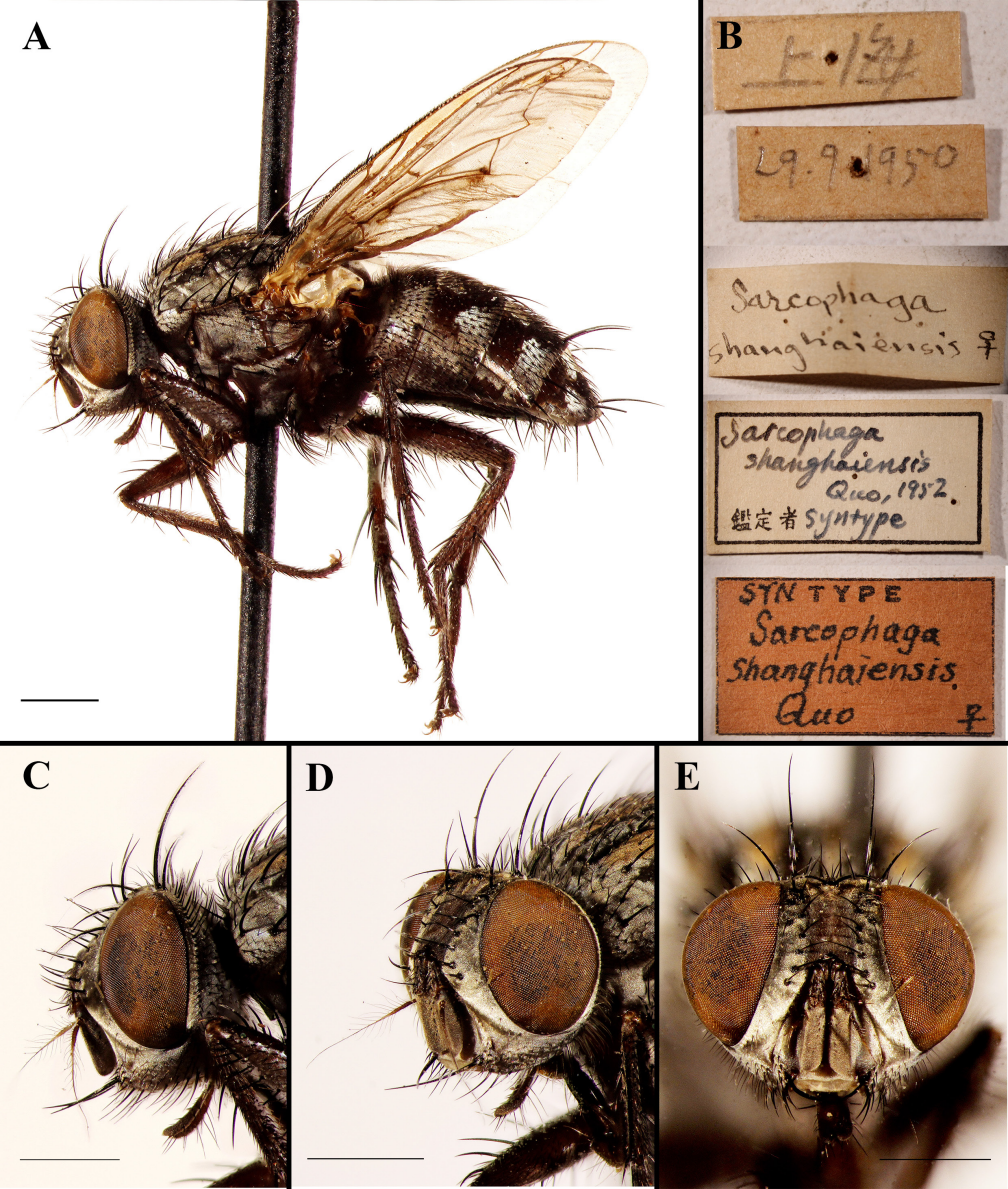
Syntype of *Sarcophaga shanghaiensis* Quo, 1952, female. **A**. Body, lateral view; **B**. Labels; **C**. Head, lateral view; **D**. Head, anterolateral view; **E**. Head, anterior view. Scale bars: 1 mm.

**Figure 22.**
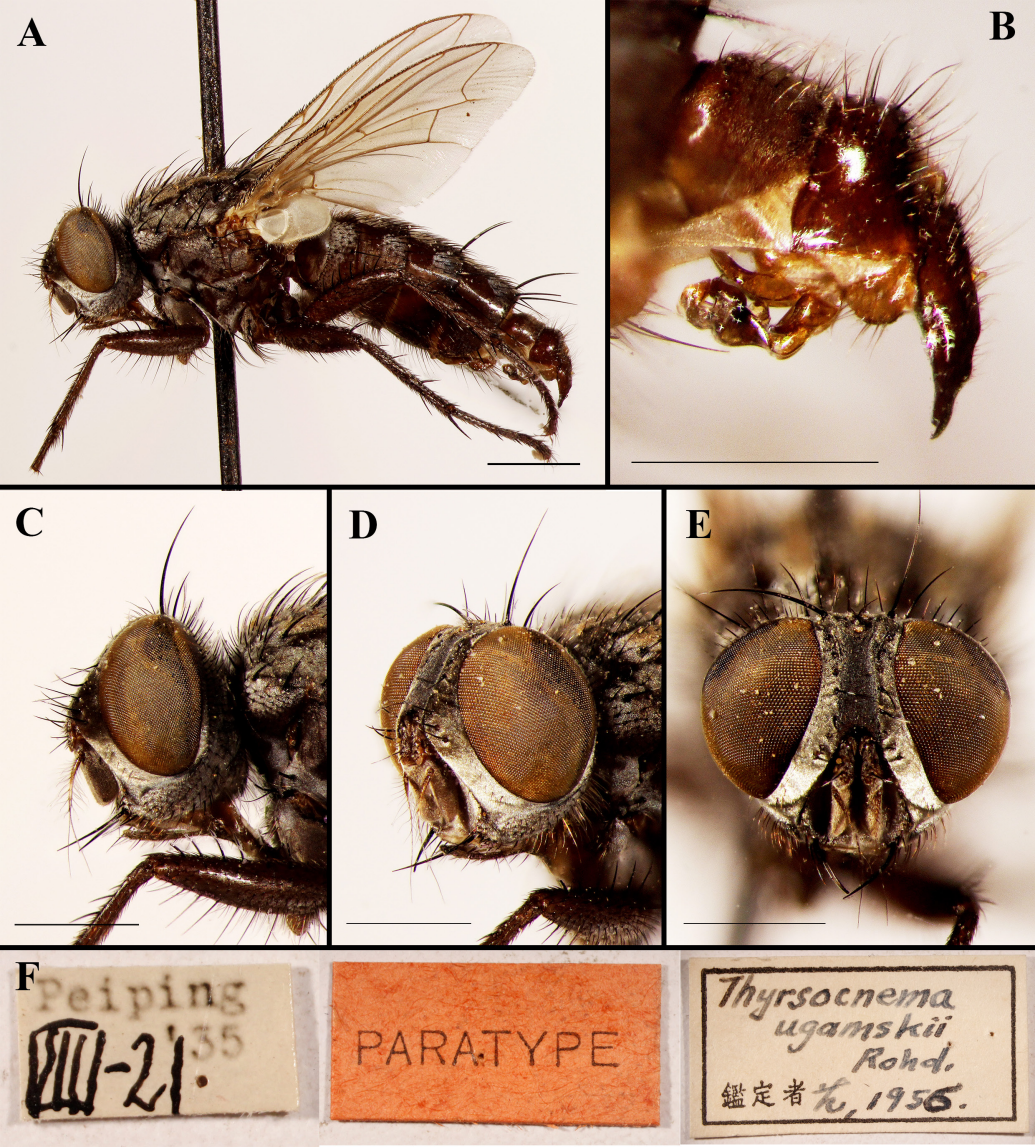
Paratype of *Sarcophaga shanghaiensis* Quo, 1952, male. **A**. Body, lateral view; **B**. Terminalia, lateral view; **C**. Head, lateral view; **D**. Head, anterolateral view; **E**. Head, anterior view; **F**. Labels. Scale bars: 1 mm.

**Name**. *Sarcophaga shanghaiensis* Quo, 1952: 65.

**Type locality**. China: Beijing, Nanjing, and Shanghai.

**Material examined**. Syntypes (14♂♂, 3♀♀): 1 male, Peiping (Beijing), 21.viii.1935, no further data. 3 females, Shanghai, 29.ix.1950, no further data. Others: 1 male, Peiping (Beijing), 25.iv.1932, no further data; 1 male, Peiping (Beijing), 27.vi.1932, no further data; 1 male, Peiping (Beijing), 1.viii.1932, no further data; 1 male, Peiping (Beijing), 29.viii.1932, no further data; 1 male, Peiping (Beijing), 21.viii.1935, no further data; 1 male, Peiping (Beijing), 25.viii.1935, no further data; 4 males, Peiping (Beijing), no further data; 3 males, Nanjing, no further data.

**Identity**. Sarcophaga (Asiopierretia) ugamskii Rohdendorf, 1937.

**References**. Pierreta (Pseudothyrsocnema) ugamskii: Fan (1965: 232, 234, 256).

Pierreta (Asiopierretia) ugamskii: Fan (1992: 664, 680, 681), Xue and Chao (1996: 1628, 1637, 1638), Yue (2016: 41, 100).

*Pierreta ugamskii*: Wang et al. (1992: 172), Yu and Sun (1993: 424), Lei and Zhou (1998: 256), Liu et al. (2008: 71).

*Asiopierretia ugamskii*: Fan and Pape (1996: 247).

Asceloctella (Asiopierretia) ugamskii: Verves (2020: 32).

Sarcophaga (Asiopierretia) ugamskii: Pape (1996: 298).

Sarcophaga (Asiopierretia) ugamskii. Wang (2013: 27), Zhang (2014: 11, 25).

**Remarks**. The type series is composed of 16 males and 12 females, of which 14 males and 3 females were recovered in the present study. Quo (1952) did not designate a holotype, which means that the specimens are syntypes.

**18. shantungensis**

Fig. 23

**Figure 23.**
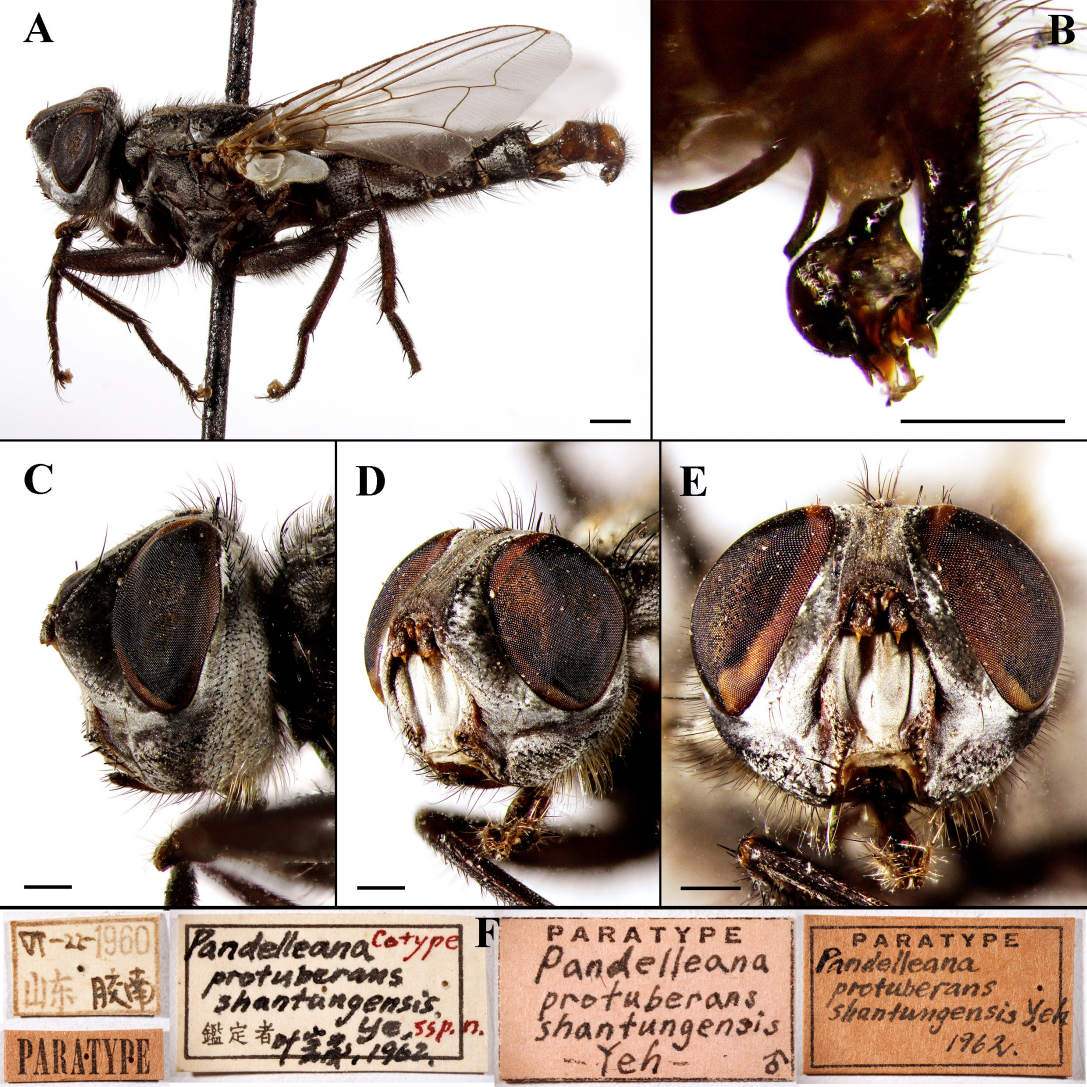
Paratype of *Pandelleana protuberans shantungensis* Ye, 1965, male. **A**. Body, lateral view; **B**. Terminalia, lateral view; **C**. Head, lateral view; **D**. Head, anterolateral view; **E**. Head, anterior view; **F**. Labels. Scale bars: 1 mm.

**Name**. *Pandelleana protuberans shantungensis* Ye, 1965: 302.

**Type locality**. China: Shandong, Ao Shan Wei.

**Material examined**. Paratypes: 2 ♂♂, Shandong, Jiaonan, 22.vi.1960, no further data.

**Identity**. Sarcophaga (Pandelleana) protuberans Pandellé, 1896.

**References**. *Pandelleana protuberans protuberans*: Fan (1965: 228, 229), Fan (1992: 646, 647).

*Pandelleana protuberans shantungensis*: Fan (1965: 229), Fan (1992: 646, 647), Xue and Chao (1996: 1605, 1607), Zhang (2014: 9).

Sarcophaga (Pandelleana) protuberans: Xue and Chao (1996: 1605, 1606), Fan and Pape (1996: 253), Pape (1996: 371), Wang (2013: 34), Zhang (2014: 9), Zhang (2014: 18, 48).

*Pandelleana shantungensis* Verves (2020: 36).

**Remarks**. The type series is composed of a holotype and two paratypes, all males. The original paper does not state the depository. Only the two paratypes were recovered in the present study.

**19. *shenzhenensis***

**Name**. *Beziella shenzhenensis* Fan, 2002: 92.

**Type locality**. China: Guangdong, Shenzhen.

**Material examined**. None.

**Identity**. Sarcophaga (Beziella) shenzhenensis (Fan, 2002).

**References**. Myorhina (Pachystyleta) shenzhenensis: Verves (2020: 36);

*Sarcorohdendorfia shenzhenensis*: Verves (2020: 54).

**Remarks**. The type series is composed of a single specimen, the male holotype by original designation. A careful search for the holotype in the collection was unsuccessful.

**20. *shuxia***

Fig. 24

**Figure 24.**
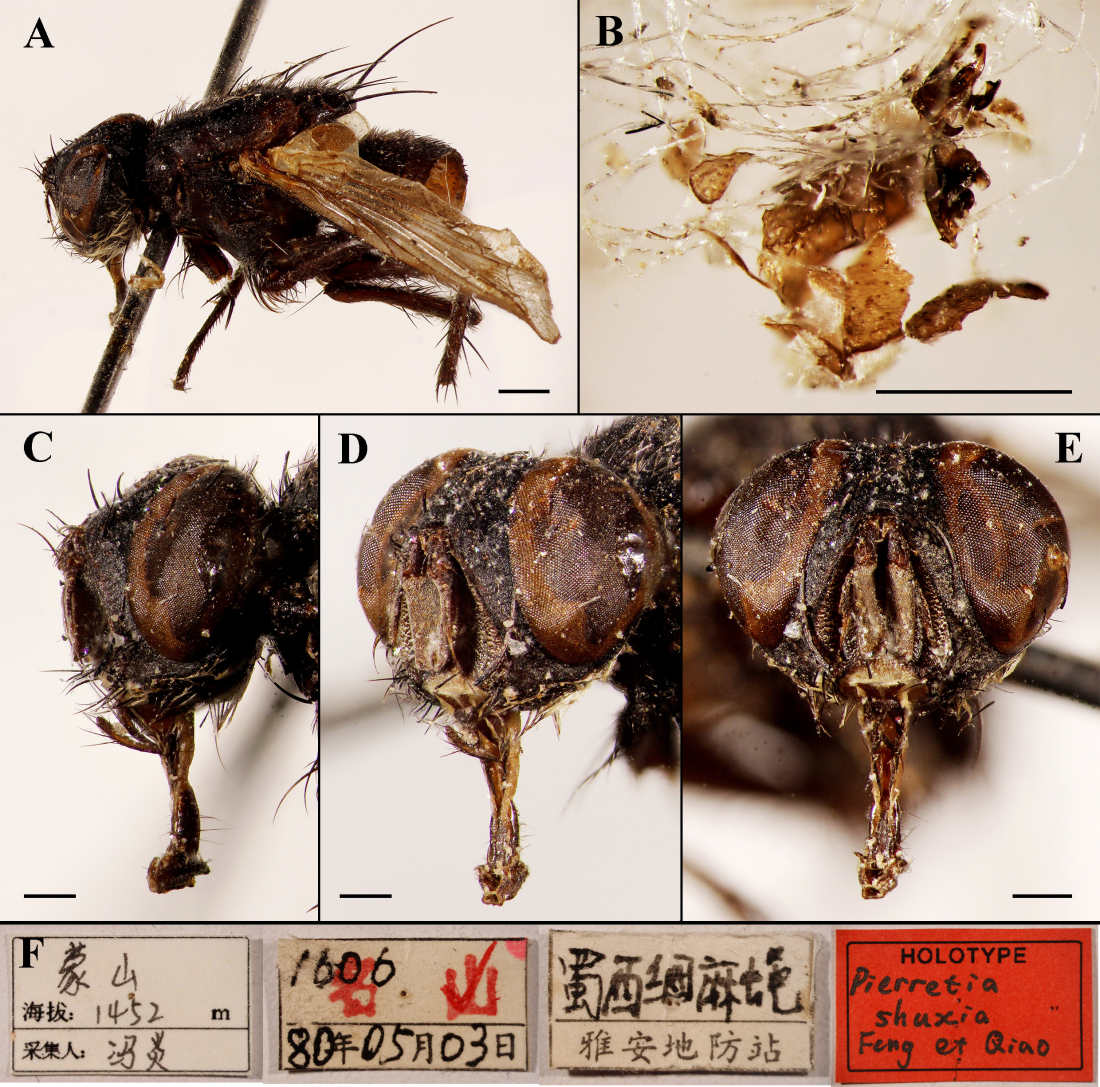
Holotype of *Pierretia shuxia* Feng & Qiao, 2003, male. **A**. Body, lateral view; **B**. Terminalia, lateral view; **C**. Head, lateral view; **D**. Head, anterolateral view; **E**. Head, anterior view; **F**. Labels. Scale bars: 1 mm.

**Name**. *Pierretia shuxia* Feng & Qiao, 2003: 268.

**Type locality**. China: Sichuan, Mingshan Mountains, Mount Meng.

**Material examined**. Holotype (♂): Sichuan, Mingshan Mountains, Mount Meng, 1452 m, 3.v.1980, Yan Feng leg.

**Identity**. Sarcophaga (Bellieriomima) shuxia (Feng & Qiao, 2003).

**References**. *Pierretia shuxia*: Zhang (2014: 9).

*Povolnymyia shuxia*: Verves (2020: 37).

Sarcophaga (Bellieriomima) shuxia: Wang (2013: 28), Zhang (2014: 11, 26).

**Remarks**. The type series is composed of a single specimen, the holotype by original designation.

**21. *spatulifera***

Fig. 25

**Figure 25.**
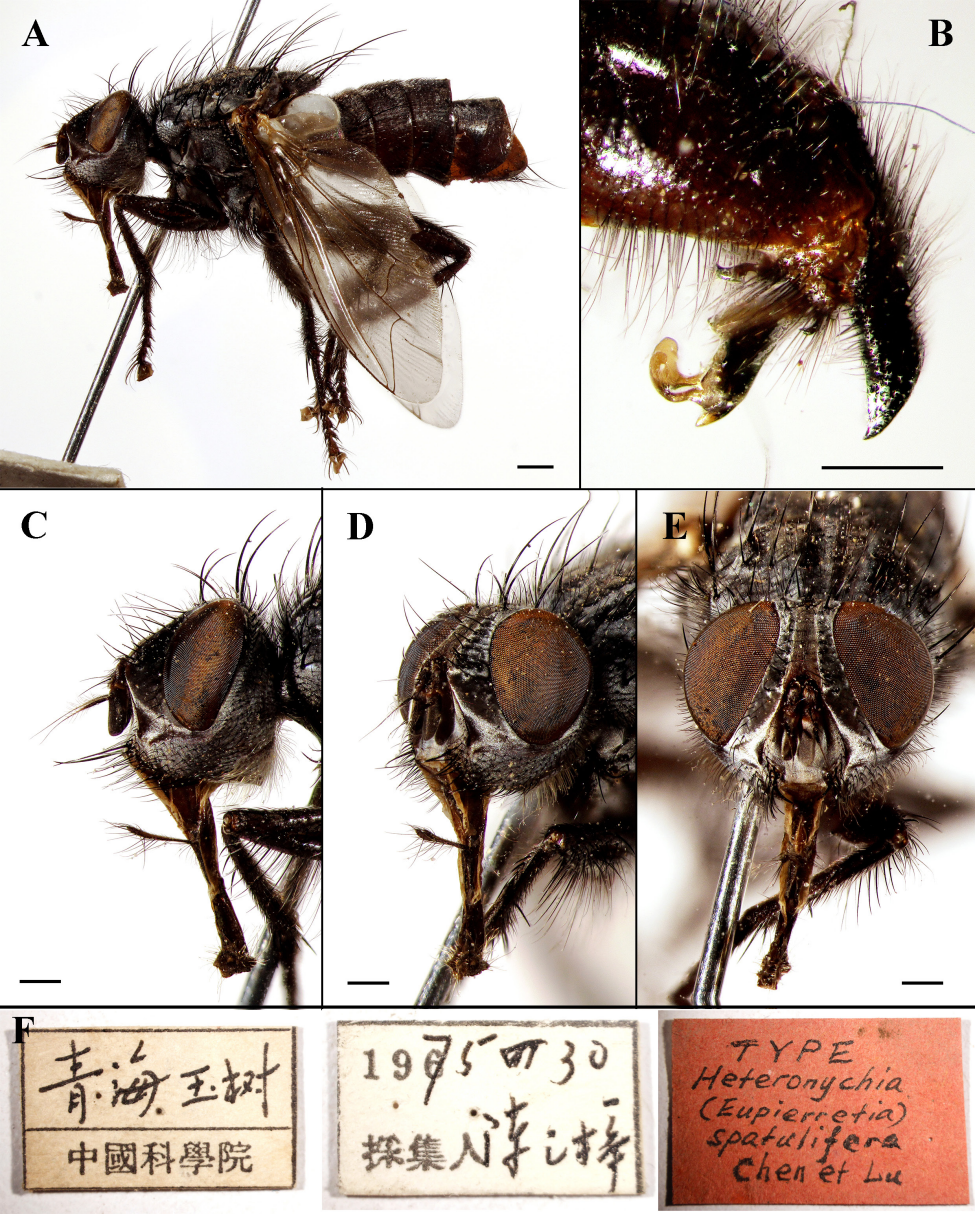
Holotype of Heteronychia (Eupierretia) spatulifera Chen & Lu, 1981, male. **A**. Body, lateral view; **B**. Terminalia, lateral view; **C**. Head, lateral view; **D**. Head, anterolateral view; **E**. Head, anterior view; **F**. Labels. Scale bars: 1 mm.

**Name**. Heteronychia (Eupierretia) spatulifera Chen & Lu, 1981: 256.

**Type locality**. China: Qinghai, Yushu.

**Material examined**. Holotype (♂): Qinghai, Yushu, 30.vi.1975, Zhizi Chen Leg.

**Identity**. Sarcophaga (Heteronychia) curvifemoralis (Li, 1981).

**References**. *Heteronychia* (s. str.) *spatulifera* Fan (1992: 655, 656), Zhang (2014: 9).

*Heteronychia spatulifera*: Xue and Chao (1996: 1593, 1594, 1597), Fan and Pape (1996: 250), Xue and Wang (2006: 228 Chen and Lu), Whitmore (2010: 58).

Sarcophaga (Heteronychia) spatulifera: Pape (1996: 334), Wang (2013: 30), Zhang (2014: 33).

Sarcophaga (Heteronychia) curvifemoralis: Whitmore (2011: 26).

**Remarks**. The type series is composed of seven males with one holotype and all were not recovered.

**22. *tsinanensis***

Fig. 26

**Figure 26.**
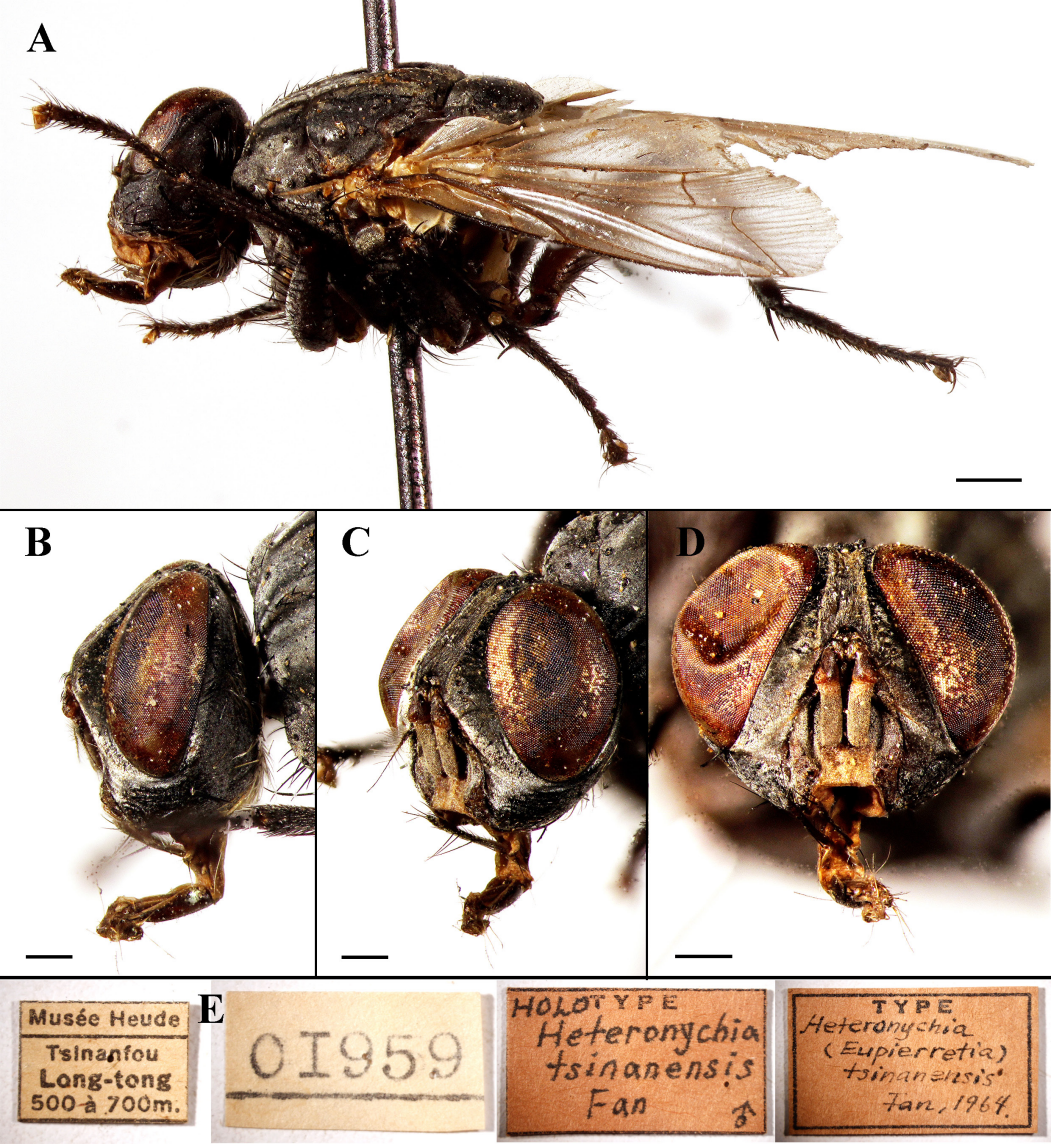
Holotype of Heteronychia (Eupierretia) tsinanensis Fan, 1964, male. **A**. Body, lateral view; **B**. Head, lateral view; **C**. Head, anterolateral view; **D**. Head, anterior view; **E**. Labels. Scale bars: 1 mm.

**Name**. Heteronychia (Eupierretia) tsinanensis Fan, 1964: 314.

**Type locality**. China: Shandong, Tsinan.

**Material examined**. Holotype (♂): Shandong, Jinan, no further data.

**Identity**. Sarcophaga (Heteronychia) tsinanensis (Fan, 1964).

**References**. Heteronychia (Eupierretia) tsinanensis: Fan (1965: 240, 241), Chao and Zhang (1985: 132);

*Heteronychia* (s. str.) *tsinanensis*): Fan (1992: 654), Verves (2020: 30).

*Heteronychia tsinanensis*: Shi (1993: 510), Fan and Pape (1996: 250), Xue and Chao (1996: 1593, 1594, 1597);

Sarcophaga (Heteronychia) tsinanensis: Pape (1996: 335), Whitmore (2010: 124), Wang (2013: 30), Zhang (2014: 14, 33).

**Remarks**. The type series is composed of a single specimen, the holotype by original designation. The holotype has been dissected, but the terminalia were not recovered. Fan (1964: 319) wrote that the holotype was “a unique male specimen whose head and thorax have been lost”. Searching for the male abdomen and terminalia gave no result.

**23. *tsushimae***

Fig. 27

**Figure 27.**
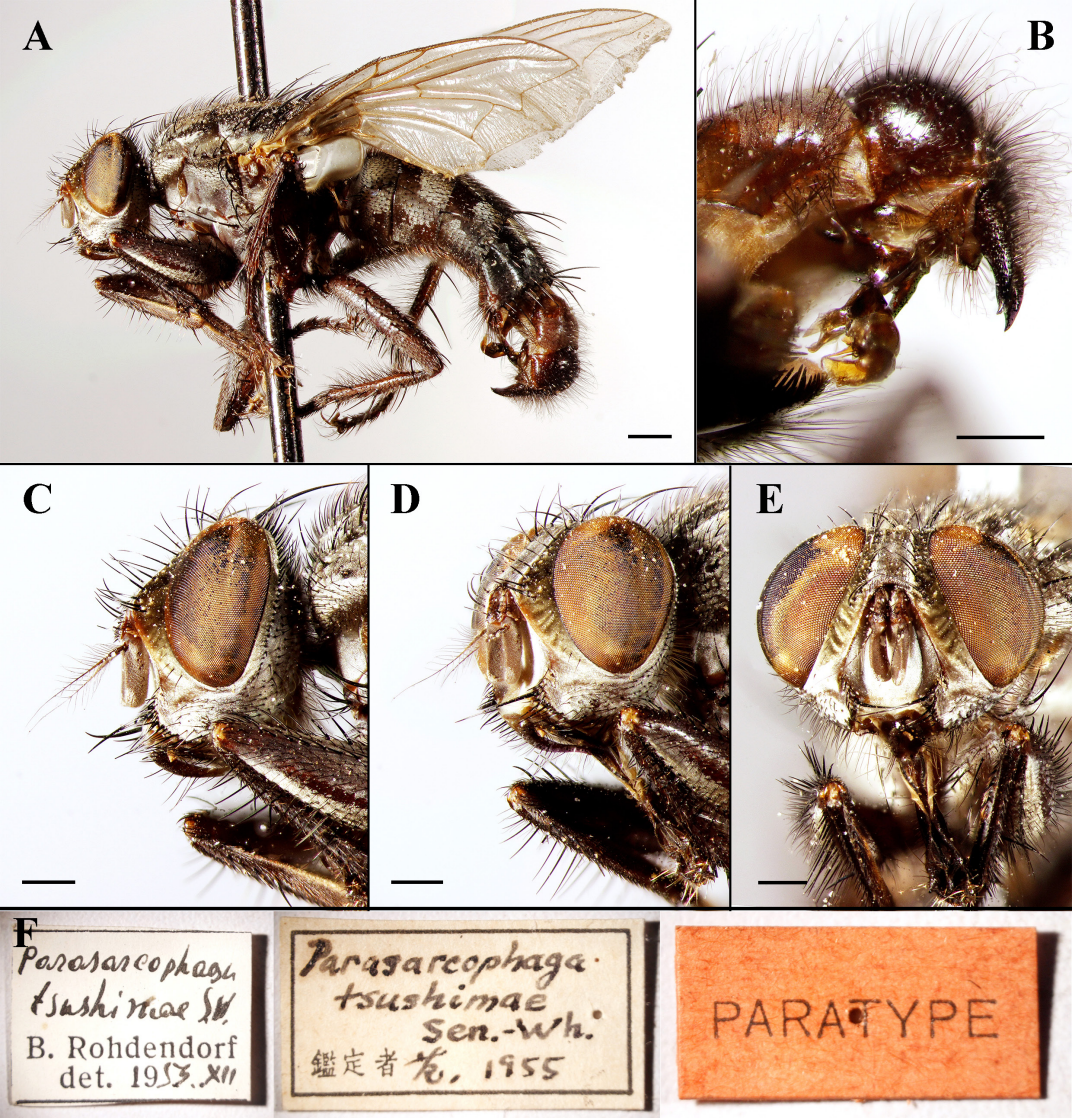
Paratype of *Sarcophaga tsushimae* Senior-White, 1924, male. **A**. Body, lateral view; **B**. Terminalia, lateral view; **C**. Head, lateral view; **D**. Head, anterolateral view; **E**. Head, anterior view; **F**. Labels. Scale bars: 1 mm.

**Name**. *Sarcophaga tsushimae* Senior-White, 1924: 248.

**Type locality**. Japan, Tsushima Strait, Iki I.

**Material examined**. Two specimens (♂♂), labelled as paratypes, Nanjing, no further data.

**Identity**. Sarcophaga (Liosarcophaga) tsushimae Senior-White, 1924.

**References**. *Pandelleisca tsushimae*: Fan and Pape (1996: 253).

Sarcophaga (Liosarcophaga) tsushimae: Pape (1996: 359).

**Remarks**. The type series is composed of a single specimen, the male holotype by monotypy. Senior-White (1924: 248) explicitly stated that “The unique type is in the Austrian State Museum, 1♂, Iki Id., Straits of Tsushima, September, (Fruhstorfer)”, and no further material is mentioned. Senior-White et al. (1940: 203) also referred to this single specimen. The paratype labels are evidently later additions and the specimens recovered in this study have no type status.

**24. *yunnanensis***

Fig. 28

**Figure 28.**
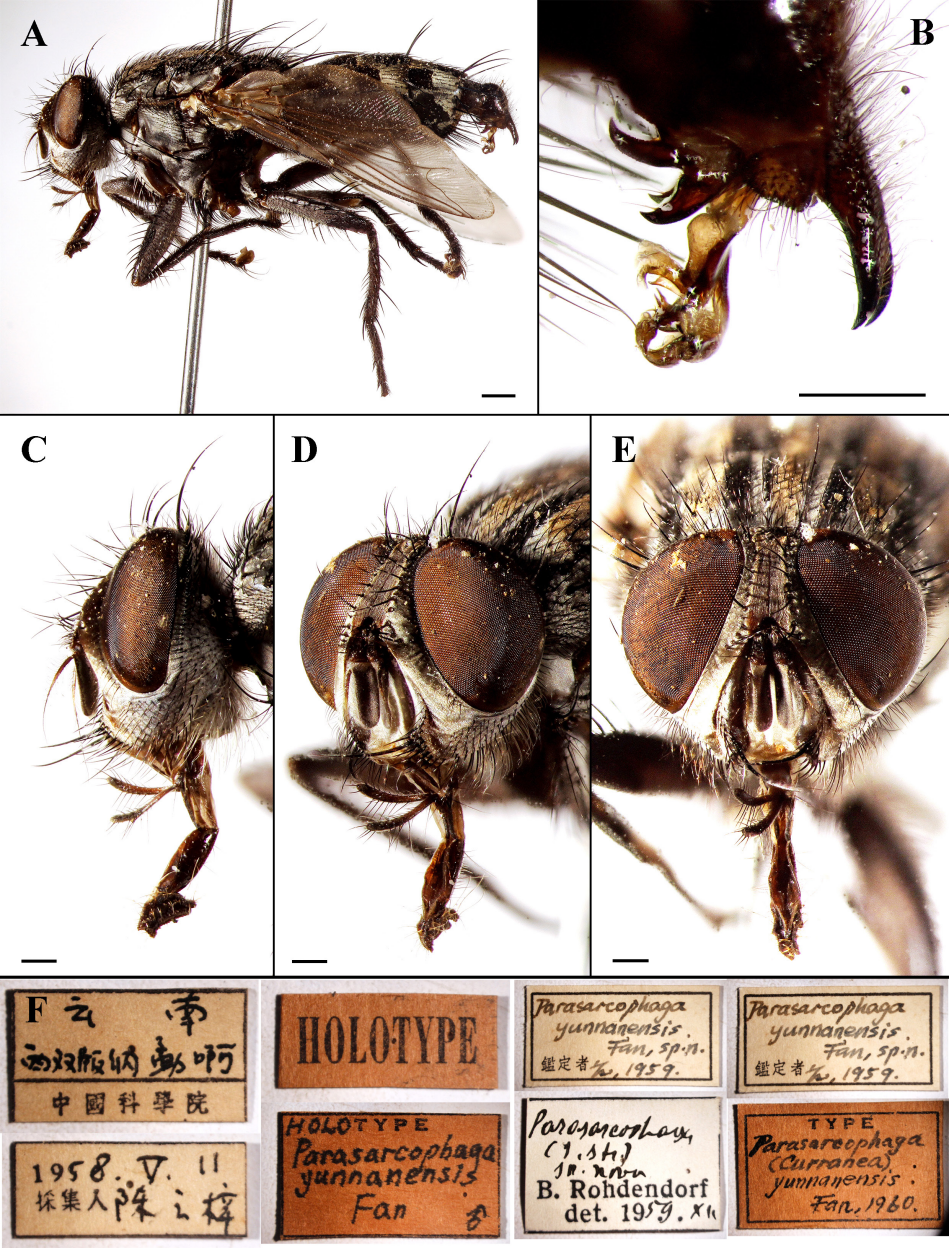
Holotype of Parasarcophaga (Curranea) yunnanensis Fan, 1964, male. **A**. Body, lateral view; **B**. Terminalia, lateral view; **C**. Head, lateral view; **D**. Head, anterolateral view; **E**. Head, anterior view; **F**. Labels. Scale bars: 1 mm.

**Name**. Parasarcophaga (Curranea) yunnanensis Fan, 1964: 308.

**Type locality**. China: Yunnan, Shishong-Baanna, Men-ah.

**Material examined**. Holotype (♂): Yunnan, Xishuangbanna (Shishong-Baanna), Meng’a (Men-ah), 11.v.1958, Zhizi Chen leg.

**Identity**. Sarcophaga (Pandelleisca) yunnanensis (Fan, 1964).

**References**. Liosarcophaga (Curranea) yunnanensis: Verves (2020: 42).

*Pandelleisca yunnanensis*: Fan and Pape (1996: 253).

Parasarcophaga (Curranea) yunnanensis: Fan (1965: 276, 277), Xue and Chao (1996: 1609, 1620, 1624).

Sarcophaga (Pandelleisca) yunnanensis: Pape (1996: 373).

**Remarks**. The type series is composed of the male holotype and three male paratypes. Only the holotype was recovered in this study.

**25. *xizangensis***

Fig. 29

**Figure 29.**
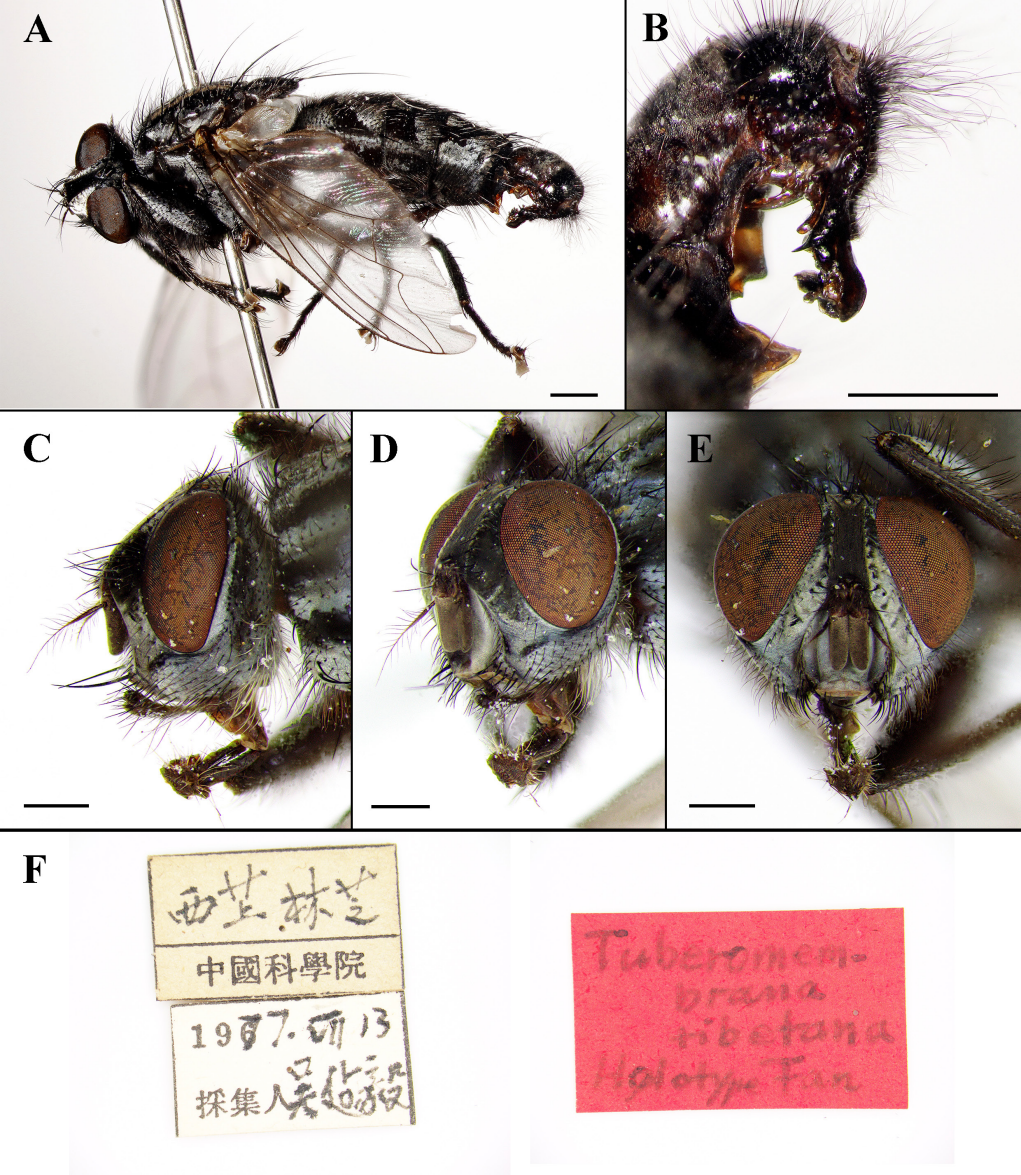
Holotype of *Tuberomembrana xizangensis* Fan, 1981, male. **A**. Body, lateral view; **B**. Terminalia, lateral view; **C**. Head, lateral view; **D**. Head, anterolateral view; **E**. Head, anterior view; **F**. Labels. Scale bars: 1 mm.

**Name**. *Tuberomembrana xizangensis* Fan, 1981: 314.

**Type locality**. China: Xizang, Nyinchi.

**Material examined**. Holotype (♂): Xizang, Linzhi (Nyinchi), 13.vii.1977, Jianyi Wu leg.

**Identity**. Sarcophaga (Tuberomembrana) xizangensis (Fan, 1981).

**References**. *Tuberomembrana xizangensis*: Huang (1988: 575), Fan (1992: 647, 648), Xue and Chao (1996: 1654, 1656), Fan and Pape (1996: 257), Xue and Wang (2006: 236), Yue (2016: 27, 80), Verves (2020: 39).

Sarcophaga (Tuberomembrana) xizangensis: Pape (1996: 412), Wang (2013: 38), Zhang (2014: 23, 62).

**Remarks**. The type series is composed of a holotype plus four paratypes, all males. Only the holotype was recovered in the present study.

## Additional information

### Artificial Intelligence (AI) use

The authors accept full responsibility for the content of the manuscript, including the disclosure of any use of AI.

No AI tools were used in the preparation of this manuscript.

### Author ORCIDs

C. Wang https://orcid.org/0000-0001-7251-1860

T. Pape https://orcid.org/0000-0001-6609-0609

W. Zhu https://orcid.org/0000-0002-9607-2513

### Data availability

All of the data that support the findings of this study are available in the main text.
